# Correction: The dynamics of intonation: Categorical and continuous variation in an attractor-based model

**DOI:** 10.1371/journal.pone.0231221

**Published:** 2020-03-26

**Authors:** The PLOS ONE Staff

## Notice of republication

This article was republished on March 12, 2020, to correct formatting errors introduced during the typesetting process. The publisher apologizes for these errors. Please download this article again to view the correct version. The originally published, uncorrected article and the republished, corrected article are provided here for reference.

## Supporting information

S1 File Originally published, uncorrected article. (PDF)

S2 File Republished, corrected article. (PDF)

## References

[1] Roessig S, Mücke D, Grice M (2019) The dynamics of intonation: Categorical and continuous variation in an attractor-based model. PLoS ONE 14(5): e0216859 10.1371/journal.pone.0216859 31120891 PMC6532892

