# Supplementary material for: The dynamics of intonation: Categorical and continuous variation in an attractor-based model
Source: PLoS One. 2019 May 23;14(5):e0216859. doi: 10.1371/journal.pone.0216859 (PMC6532892; doi:10.1371/journal.pone.0216859)
Supplement: S1 Table — (PDF) [file pone.0216859.s001.pdf]

Nohme	Mohme	Bohme	Lohne	Wohme
Nohse	Mohwe	Bohwe	Lohle	Wohse
Nahne	Mahne	Bahle	Lahse	Wahne
Nahle	Mahse	Bahwe	Lahle	Wahwe

S1 Table. Target words used in the experiment (all nonce words).
