# Supplementary material for: The dynamics of intonation: Categorical and continuous variation in an attractor-based model
Source: PLoS One. 2019 May 23;14(5):e0216859. doi: 10.1371/journal.pone.0216859 (PMC6532892; doi:10.1371/journal.pone.0216859)
Supplement: S2 Table — (PDF) [file pone.0216859.s002.pdf]

Amboss	*Anvil*
Besen	*Broom*
Bohrer	*Drill*
Bürste	*Brush*
Hammer	*Hammer*
Pinsel	*Paint brush*
Rolle	*Paint roller*
Säge	*Saw*
Schere	*Scissors*
Zange	*Pliers*

S2 Table. Tools used in the experiment with English translation.
