# Supplementary figures and images for: The dynamics of intonation: Categorical and continuous variation in an attractor-based model
Source: PLoS One. 2019 May 23;14(5):e0216859. doi: 10.1371/journal.pone.0216859 (PMC6532892; doi:10.1371/journal.pone.0216859)

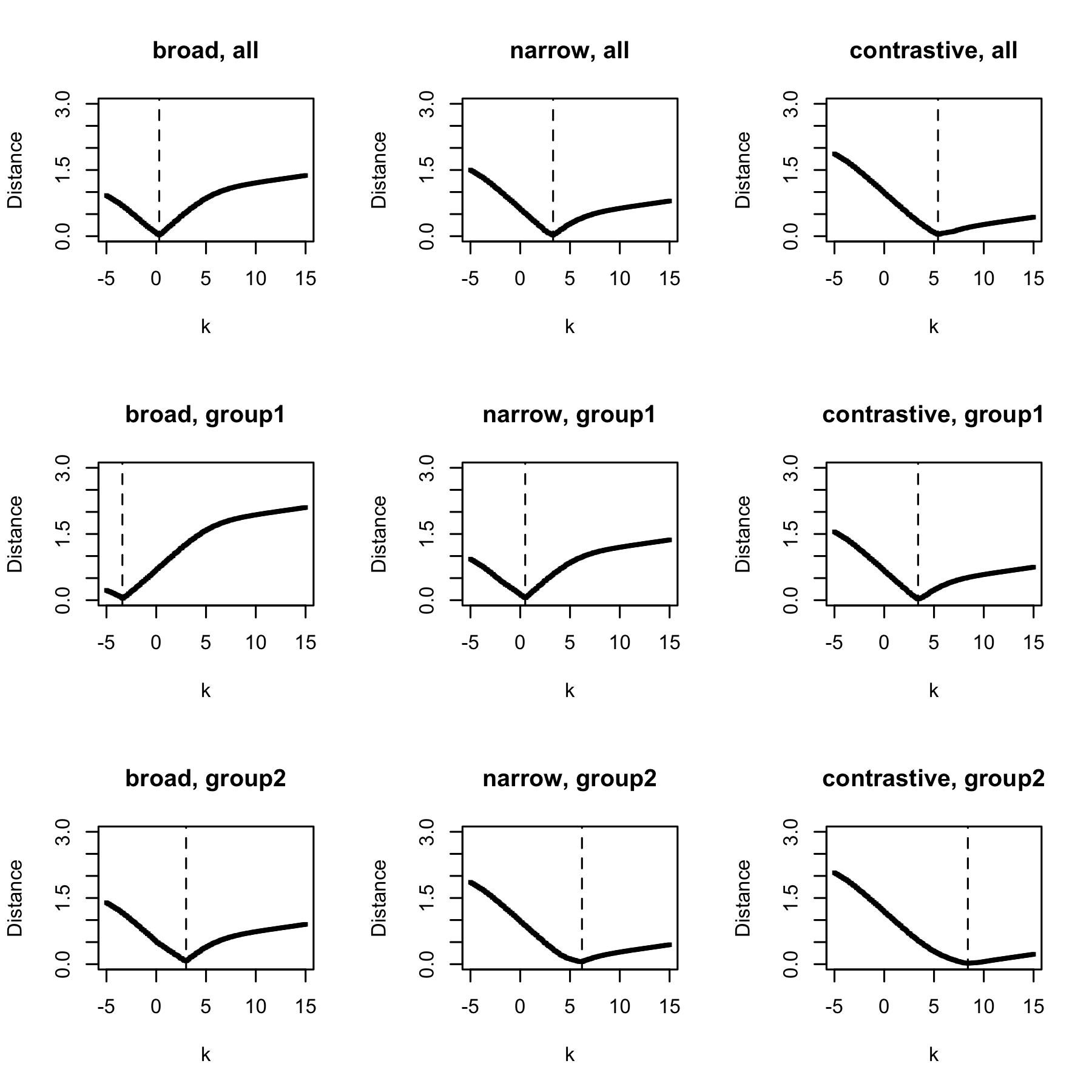

Supplement: S1 Fig — The dashed vertical line represents the k value with minimum distance. (TIFF) [file pone.0216859.s003.tiff]
