# Supplementary material for: Correction: The dynamics of intonation: Categorical and continuous variation in an attractor-based model
Source: PLoS One. 2020 Mar 26;15(3):e0231221. doi: 10.1371/journal.pone.0231221 (PMC7098631; doi:10.1371/journal.pone.0231221)
Supplement: S2 File — (PDF) [file pone.0231221.s002.pdf]

RESEARCH ARTICLE

The dynamics of intonation: Categorical and continuous variation in an attractor-based model

Simon Roessig*, Doris Mücke, Martine Grice

IfL–Phonetics, University of Cologne, Cologne, Germany

* mail@simonroessig.de

Check for updates

OPEN ACCESS

Citation: Roessig S, Mücke D, Grice M (2019) The dynamics of intonation: Categorical and continuous variation in an attractor-based model. PLoS ONE 14(5): e0216859. https://doi.org/10.1371/journal.pone.0216859

Editor: Kristof Strijkers, CNRS - Université d'Aix-Marseille, FRANCE

Received: October 2, 2018

Accepted: April 30, 2019

Published: May 23, 2019

Copyright: © 2019 Roessig et al. This is an open access article distributed under the terms of the Creative Commons Attribution License, which permits unrestricted use, distribution, and reproduction in any medium, provided the original author and source are credited.

Data Availability Statement: The data underlying this study are available from https://doi.org/10.5281/zenodo.2611316 (DOI: 10.5281/zenodo.2611316).

Funding: Funding was provided by the Deutsche Forschungsgemeinschaft (CRC1252 Prominence in Language, Project A04) to DM, http://www.dfg.de. The funders had no role in study design, data collection and analysis, decision to publish, or preparation of the manuscript.

Competing interests: The authors have declared that no competing interests exist.

Abstract

The framework of dynamical systems offers powerful tools to understand the relation between stability and variability in human cognition in general and in speech in particular. In the current paper, we propose a dynamical systems approach to the description of German nuclear pitch accents in focus marking to account for both the categorical as well as the continuous variation found in intonational data. We report on results from 27 native speakers and employ an attractor landscape to represent pitch accent types in terms of f0 measures in a continuous dimension. We demonstrate how the same system can account for both the categorical variation (relative stability of one prosodic category) as well as the continuous variation (detailed modifications within one prosodic category). The model is able to capture the qualitative aspects of focus marking such as falling vs. rising pitch accent types as well as the quantitative aspects such as less rising vs. more rising accents in one system by means of scaling a single parameter. Furthermore, speaker group specific strategies are analysed and modelled as differences in the scaling of this parameter. Thus, the model contributes to the ongoing debate about the relation between phonetics and phonology and the importance of variation in language and speech.

1. Introduction

The question of how phonology, with its categorical representations, and phonetics, with its continuous signals, relate to each other has been a central topic of debate in recent years. It has long been the prevalent view that mental representations of speech sounds are best conceptualised as symbols and discrete rules or constraints operating on these symbols, and that the result of this discrete computation is translated into a continuous signal, i.e. an acoustic output produced via articulatory movements. However, there is accumulating evidence that the categorical and continuous aspects of speech are deeply intertwined, and many researchers have suggested that it is fruitful to think about how continuous traits can be incorporated into our models of mental representations, e.g. [1–7]. This view complements a more general trend in the cognitive sciences that dispenses of the computer metaphor of the mind [8–11] and acknowledges the continuity of cognitive processes [12–14].

One of the main motivations in the fields of phonetics and phonology for integrating categorical and continuous representations is that a great deal of detailed variation is processed in the production and perception of sound patterns. This variation is ubiquitous across many aspects of speech and constitutes a significant part of the memory of a speaker. The important role of variation in both production and perception indicates that mental representations of phonological forms are continuous in nature and/or extremely detailed [1], aspects that symbolic abstractionist models are not able to capture. An interesting example is the phenomenon known as incomplete neutralisation where subtle acoustic differences between voiceless and devoiced final obstruents have been reported, e.g. [15–18]. These subtle but robust differences cannot be accounted for by a purely categorical approach. Hence, continuous phenomena do not only play a role in the realisation of sounds, but categorical abstractions and continuous details have to be integrated to capture the complexities of the cognitive side of speech production and perception.

In the domain of prosody, it has been a long-term endeavour to identify the categorical and continuous components of intonation. One of the greatest difficulties in this quest is that the native speakers' intuitions about intonational contrasts and their associated meanings are not as clear as they are for lexical contrasts [19]. This situation makes it hard to distinguish between categorical and continuous uses of phonetic dimensions. For example, a nuclear pitch accent–the part of the pitch contour on and around the most prominent word in the phrase–can be characterised by the relative height of its f0 peak in comparison to the immediate context, a phonetic dimension also known as *target height*. This dimension plays a role in the distinction of pitch accent types, e.g. for H* and L+H* in the transcription system ToBI for English [20,21], but is also reported to be used in a gradient way to achieve prosodic prominence associated with emphasis [22] and contrastive focus [23]. If the phonetic differences are small and subject to gradient variation, it is difficult to argue that they should be represented as different accent categories. Although many transcription systems maintain pitch accent types with subtle differences, like H* and L+H* in the aforementioned ToBI [20] and GToBI [24], the uncertainty associated with the distinction is reflected in higher inter-transcriber disagreements for these pitch accent types [21]. Again, we can observe that the boundary between what can be considered strictly categorical (interpreted as pitch accent type), and what can be described as continuous (interpreted as phonetic detail) is blurred. Rather, the encoding of communicative functions appears to involve both categorical and continuous uses of the same phonetic dimensions.

In addition to variability within categories, there is also a great deal of variation in the frequency of occurrence of these categories, both across different speakers as well as within the same speaker. For example, one pragmatic function might be expressed by one intonation contour type most of the time, but can also be encoded by an alternative intonation contour type in a minority of cases. Likewise, the same pitch accent type might be used for multiple functions. These findings have motivated a distributional view, where statistical tendencies of form-function mapping have been attested (for English: [25,26]; for German: [27,28]; for Italian: [29,30]). The idea that distributions are essential for phonological models has also been incorporated into probabilistic versions of Optimality Theory [31,32] which hypothesise statistical patterns of different constraint rankings or numerically weighted constraints. In general, the finding that the mapping between form and function appears to be probabilistic is in line with the aforementioned hypothesis that the mind works in a continuous manner and simultaneously passes through multiple states that have different probabilities of being the state with the highest activation [12].

In sum, we can identify two types of variation: *continuous variation* characterised by detailed phonetic differences within one phonological category and *categorical variation*

characterised by a probabilistic mapping between categories and functions. These terms are closely related to the concepts of "physical gradience" and "statistical gradience" coined by [33] referring to [34]. In his discussion of the two types of variation, [33] states that, at least in prosody, the two types of variation are often difficult to distinguish, as both are frequently used to achieve the same linguistic goals and even occur in symbiosis. An example of how continuous and categorical variation go hand in hand is the study of [35] that investigates the distribution and realisation of pitch accents. The results suggest that the mapping between focus types and pitch accent types is not one-to-one, as already reported in [27]. Instead, there are general tendencies for certain pitch accent types to occur more frequently with certain focus types, for example H+!H* accents with broad focus, H* accents with narrow focus and L+H* accents with contrastive focus. Fig 1 (adapted from [35]) shows examples of f0 contours of the three pitch accent types from this study annotated by trained labellers: In the case of H+!H* (top), the f0 contour reaches its maximum before the accented syllable (shaded area) and falls throughout the syllable. In the case of H* (middle) and L+H* (bottom), the contour rises throughout the syllable. The rising movement is shallower in H* compared to L+H*. [35] show that there is variation in the phonetic parameters within each pitch accent category and that this variation is related to focus types. Importantly, variation within the boundaries of the categories appears to mimic variation across category boundaries: For example, some speakers primarily use the shallower H* in narrow focus and the more rising L+H* in contrastive focus. Others use H* for both functions, but increase the magnitude of the pitch movement in contrastive focus as compared to narrow focus.

It is exactly this symbiosis of the two types of variation that the framework of nonlinear dynamical systems is particularly well suited to capture. While dynamical systems are completely continuous, they usually have stable states, called attractors, which can be considered as analogues to phonological categories [5,6,36–38]. Yet, these categories are directly implemented in the phonetic space. A dynamical system can have more than one attractor and varying attractor strengths, accounting for the fact that the system produces probabilistic categorical variation. In other words: The system might settle in one stable state most of the time but still be able to settle in another stable state in a minority of cases. Furthermore, it can exhibit variation around the attractors as well as modifications of the attractors, leading to continuous variation. Another interesting property of dynamical systems is that subtle, continuous variation in the parameters defining the system can introduce a qualitative change in the attractor landscape: A small adjustment in the parameters can cause an attractor to vanish or another to emerge [6]. Crucially, dynamical systems can be used to model sound patterns of language without requiring a strict separation of phonetics and phonology. Hence, there is no need for a translation or interface between the two [5,7]. Instead, phonetics and phonology are conceptualised as a single system and can thus be modelled in a single formal language. In this formal language, both the categorical and the continuous are direct outcomes of the system. The use of dynamical systems appears natural considering the endeavours of many researchers throughout the last years to account for the observation that phonological categories and fine phonetic detail are closely interrelated.

An example for a successful application of dynamical systems theory to problems in phonetics and phonology is the model of [6]. Their approach shows how one system can account for continuous variation by slight shifts of the attractors, as in the case of incomplete neutralisation in German, and for the influence of continuous variation on categorical variation by tilts of the attractor landscape, as in the case of vowel harmony in Hungarian. Another interesting example is the work of [39] who applies attractor landscapes to prosody in second language acquisition. The process of acquiring a prosodic pattern is construed as the development of an attractor landscape. By using this model, it becomes clear how categories gradually

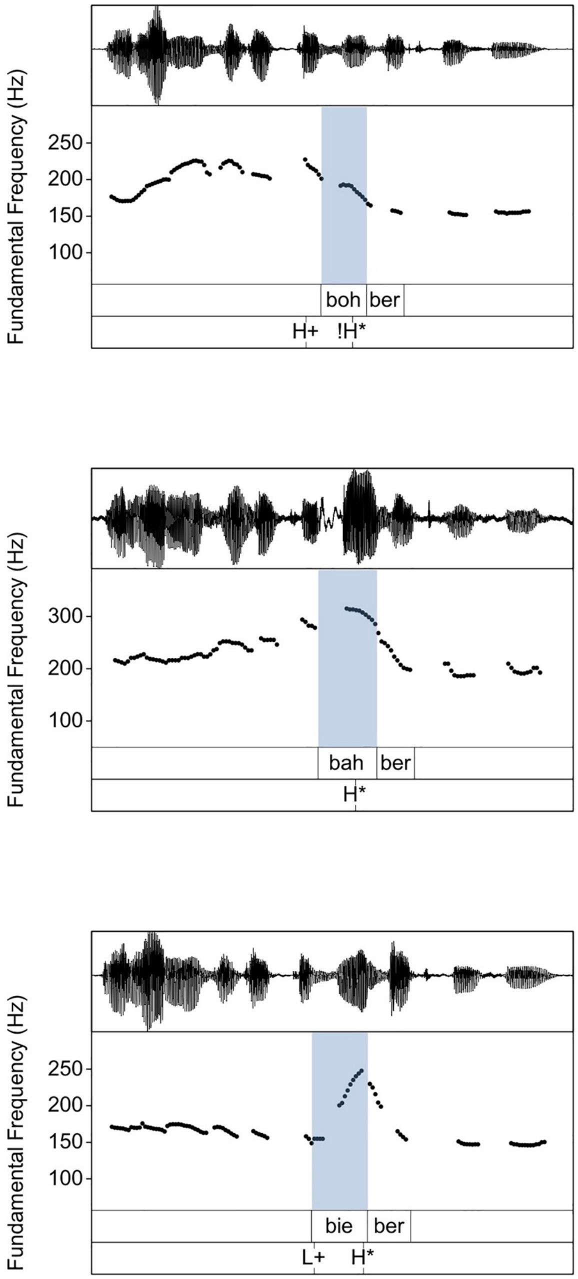

Fig 1. Examples of f0 contours: Three different nuclear pitch accent types from [35]. Top: Falling accent H+!H*. Middle: Slightly rising accent H*. Bottom: Rising accent L+H*. The shaded area marks the accented syllable of the target word.

https://doi.org/10.1371/journal.pone.0216859.g001

emerge in the learner's language and why the interlanguages of learners with different proficiency levels exhibit different proportions of categorical realisations.

The current study proposes a dynamical systems approach to the description of German nuclear pitch accents in focus marking to capture both the categorical as well as the continuous variation found in the data. The paper aims at contributing to the ongoing debate about the relation between phonetics and phonology and the importance of variation in language and

speech. It pays tribute to the broader perspective of the mind as a continuous system in which fuzzy, graded boundaries are the rule rather than the exception [12].

The rest of the paper is structured as follows: Section 2 gives an introduction into dynamical systems and attractors. Section 3 presents a large data set on German focus marking elicited in an interactive production study and gives a modelling account of the intonation data in a dynamical system. This section also sheds light on speaker-specific differences and how they can be accounted for in the outlined modelling approach. Section 4 discusses the experimental results and the modelling, and is followed by a conclusion in section 5.

2. Dynamical systems and attractors

At the heart of dynamical systems theory lies the observation, most probably first made by Newton and Leibniz, that the world can be described in terms of differential equations [40]. Differential equations give us a powerful formal tool to determine where something is moving to, or in other words, what the state of a system will be at a point in the future. Consider the simple differential equation in (1), its graph is shown in Fig 2.

$$\mathrm{F(x)} = -\frac{\mathrm{x}}{2} \quad (1)$$

The variable x is the current state of the system. What the equation tells us is in which direction and how much the state of the system will change in the future. Suppose, for example, the current state of the system is $x_0 = -0.5$. The change can be calculated by plugging this value into the function F: $F(-0.5) = -\frac{(-0.5)}{2} = \frac{0.5}{2} = 0.25$. We can now add this value to the current state to get the next state: $x_1 = (-0.5)+0.25 = -0.25$. Next, we can use this new state to calculate the change in order to get to the following state: $F(-0.25) = -\frac{(-0.25)}{2} = \frac{0.25}{2} = 0.125$. Thus, $x_2 = (-0.25)+0.125 = -0.125$. As can be presumed from these first little numerical examples, the value of x is going to move closer and closer to zero. And indeed, this is what Fig 3 reveals: with each time step, the system gets closer to zero.

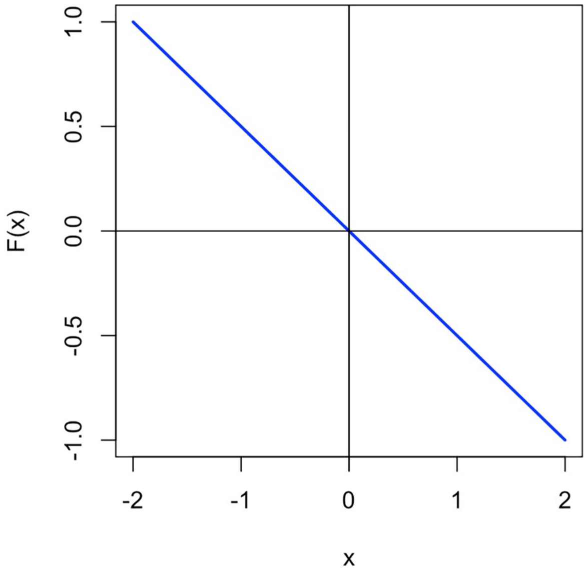

Fig 2. Graph of the function F(x) from (1).

https://doi.org/10.1371/journal.pone.0216859.g002

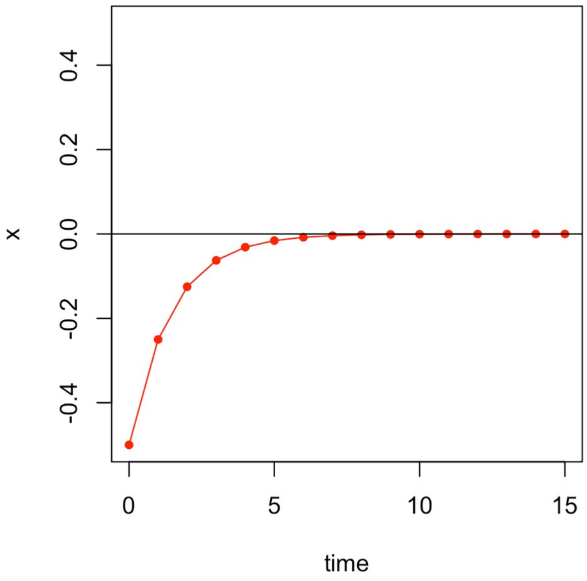

Fig 3. The evolution of the states of the system given by the differential equation in (1) starting with x_0 = -0.5.

https://doi.org/10.1371/journal.pone.0216859.g003

Fig 4 shows what happens if we start with a value of $x_0 = 0.3$, i.e. a positive value of x instead of a negative value like in the first example. In this case, the values become increasingly small, also trending towards zero–just like in the first example. The point x = 0 in this example system is called an *attractor* because it "attracts" the state of the system. The system is gravitating towards this point as time unfolds.

An attractor is a type of fixed point. Fixed points are those points of the system where the differential equation describing the system equals zero. The system in this example only has one stable state, but dynamical systems can exhibit multiple fixed points. Fixed points can be stable or unstable. While stable fixed points are called attractors, unstable fixed points are

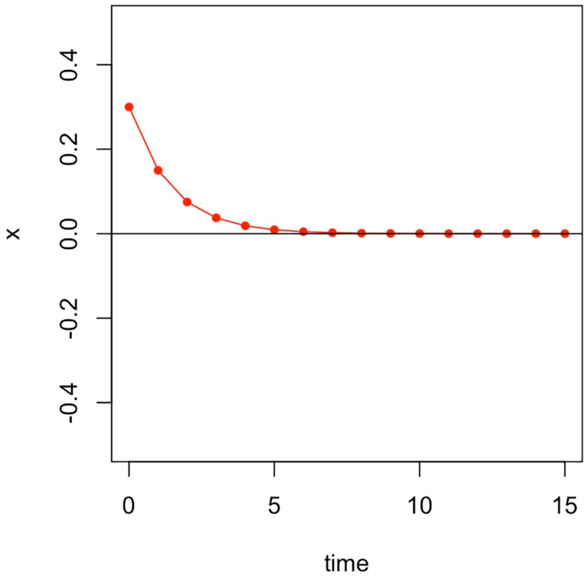

Fig 4. The evolution of the states of the system given by the differential equation in (1) starting with x_0 = 0.3.

https://doi.org/10.1371/journal.pone.0216859.g004

called repellers. Repellers exhibit the opposite effect of attractors: The system moves away from them. When we look at the graph of the differential equation in Fig 2, we can visually grasp why the system is moving towards the attractor at zero: Remember that as we move through the system we add the change in x to the current value of x to get the next state. When we start with a negative x, we get a positive value from F(x). Hence, we add a positive number to get to the next state and so x becomes larger. When we start with a positive x, we get a negative values from F(x)–so we add a negative number to get to the next state and x becomes smaller. Regardless of the first value of x, in this system all paths will continually approach zero.

The differential equation F(x) is often called the *force function* [40]. The layout of attractors, often called the attractor landscape, can also be described by the *potential energy* function which is the negative integral of the force function as given in (2) and shown in Fig 5 for our example system. Attractors are minima in the potential energy curve, in this case we can see a minimum at zero. The system will always strive to minimise the potential energy. Hence, from the perspective of the potential energy, it will move towards one of the minima. These minima are the points where the force function, the first differential of the potential energy, is zero and hence the slope of the potential is zero. A commonly used metaphor for making the notion of change towards attractors understandable more intuitively is to imagine a ball or a marble rolling through the attractor landscape [41]. In the case of the potential V(x) from (2), starting at $x_0 = -0.5$, for example, one can picture the ball rolling towards the attractor at zero. Although this metaphor is not mathematically entirely accurate, it will be used throughout our explanations to make it easier to grasp the consequences of the theoretical assumptions.

$$V(x) = \frac{x^2}{4} \tag{2}$$

Dynamical systems have been applied in the cognitive sciences and linguistics to describe a variety of phenomena, including movement coordination [42,43], categorical perception of speech sounds [36,44], learning of non-native sound patterns [45], development of cognition

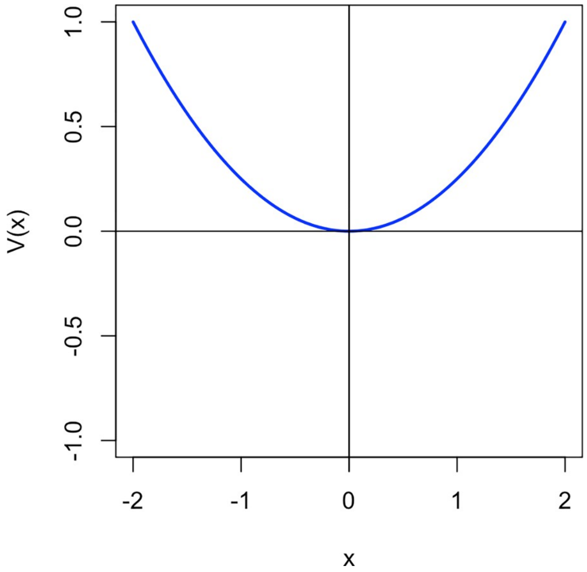

Fig 5. Graph of the potential energy function V(x).

https://doi.org/10.1371/journal.pone.0216859.g005

and action in children [46,47], and organisation of semantic knowledge [48], to mention a few (for an overview on applications in speech see [49]). One of the most widespread applications in linguistics is the approach of Articulatory Phonology [50]. In this framework, speech sounds are modelled as consisting of gestures arranged into scores to build higher forms like words, with the gestures being defined in terms of a dynamical system.

Other applications use the formal tools of dynamical systems to model the transitions between cognitive states and the competition between multiple possible states in systems that have multiple attractors. With more than one attractor, each attractor's relative strength is an important feature for the output of the system. Among others, [5,6,36] use dynamical systems with two stable states to model the contrast between two phonological categories. As opposed to a purely symbolic account, a phonological category in such a model is an attractor in a completely continuous system. Depending on how the system parameters are set, one of the attractors is assigned more strength, to account for the fact that one category is produced or perceived more frequently.

To illustrate this point, we consider the potential energy of a dynamical system that is given by the formula in (3). In this equation, the parameter k is the control parameter of the system. This parameter plays a special role in this dynamical system because the structure of the attractor landscape depends on its scaling. The graph of the potential energy of the system is given in Fig 6 with different values for k. When going from the centre, where k = 0, to the right or to the left, the system shifts from a landscape with two equally stable states to a landscape with one stable state. To return to our example of the ball rolling in the attractor landscape, imagine that there are gusts of wind from time to time that push the ball in random directions. Sometimes the gusts are weak, sometimes they are really strong. In an attractor landscape with two equally stable states (like the centre of Fig 6, where k = 0), the effect of the random gusts of wind on both states is equal. In a scenario where the attractor landscape is tilted to one side, so one of the attractors is deeper, it will be more difficult for the wind to push the ball out of this deep attractor (for example the left attractor in the left-most panel of Fig 6). While it needs a really strong gust to push the ball out of the deep attractor, only a small one is needed to push it out of the shallow opposing attractor. If in a windy environment many balls are put into the attractor landscape one after the other, the deepest attractor will collect most balls. The wind in this metaphor represents the general notion of random perturbations, or noise, in dynamical systems. The scaling of the control parameter of the system hence determines how resistant the attractors are to the influence of noise. Noise does not play a marginal role but is in fact an inherent and significant element of complex systems [51]. To determine the change in the system that has an attractor landscape like the one shown in (3), we could use the force function as given in (4). It is the negative differential of the potential, just like in the first simple example above. However, this time it is a stochastic force function where the term N represents

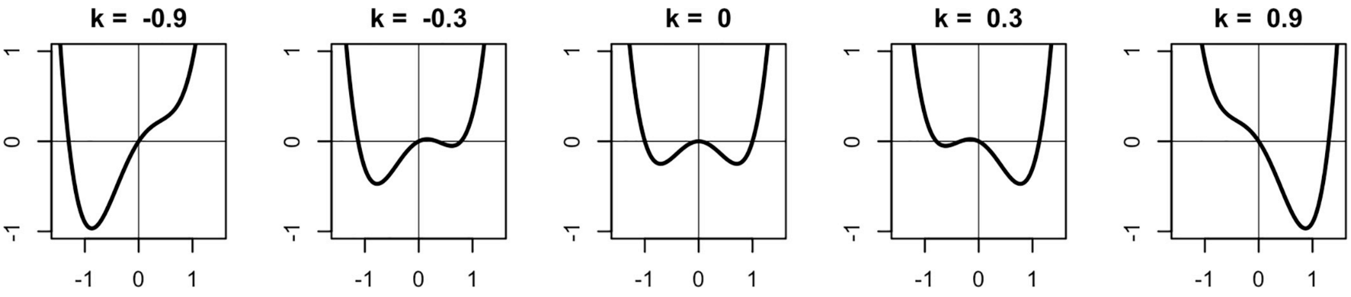

Fig 6. Attractor landscapes for the same system with different values for the parameter k.

https://doi.org/10.1371/journal.pone.0216859.g006

the influence of random noise.

$$V(x) = x^4 - x^2 - kx \quad (3)$$

$$F(x) = -(4x^3 - 2x - k) + N \quad (4)$$

Attractor models for cognitive processes emphasise the idea that the mind is best characterised as travelling on a smooth trajectory towards more or less stable states [12]. This idea is in sharp contrast to the traditional conception of the mind working like a computer that manipulates and replaces symbolic representations. During the process of settling in one stable state, the mind passes through multiple states, rather than exchanging one symbol for the other. This is why a period is expected in which categories are fuzzy and overlapping before one relatively stable state wins out over the other possible states.

However, this concept of how the mind works does not preclude the concept of the category per se, it merely updates our understanding of it. A category is not to be understood as a discrete symbol but as a stable state in the continuous landscape of the mind. But, as stated above, the trajectory might gravitate towards an attractor and not reach it entirely. Moreover, the influence of noise leads to random fluctuations in the system.

As outlined in the introduction, in the sound patterns of language, and in prosody in particular, categorical and continuous variation often work in the same direction. This finding resonates with the view of the mind as a dynamical system [12–14] and contrasts with a perspective that conceives of the continuous and categorical aspects of cognition as separate modules [8–10]. The stability and variation that a dynamical system exhibits is captured with a formal language that uses differential equations instead of symbols, rules for their manipulation and interfaces between the separate modules. When applied to the specific topic of this study, the consequence is an approach that views phonetics and phonology as parts of a single system in which phonological categories–in our case pitch accent types–are represented by the concept of attractors. Categorical variation on the one hand is explained by the relative stability of multiple attractors. Continuous variation on the other hand is ascribed to more subtle changes in the shape or location of one attractor. The general process that drives variation in the system–be they categorical or continuous–can be the same. In order to be able to shed more light on the dynamical nature of pitch accent choice and realisation, and to provide a mathematical modelling account, we conducted a production experiment yielding a large data pool. Based on these data and the thoughts outlined in the paper so far, we formulate a dynamical model of pitch accent types in German focus intonation that is able to account for the patterns found in our corpus.

The attractors in our model correspond to different pitch accent types. Their relative stability accounts for the categorical variation found in the data. In other words: If one of the attractors is stronger, the system's output will exhibit more instances of the pitch accent category associated with that attractor. However, as the relative stability of the attractors is changed, the location of the deepest point of the attractor basin shifts subtly at the same time to explain the systematic continuous variation found within one pitch accent category.

3. Experiment and simulation

3.1 Experimental methods

This study was approved by the Local Ethics Committee of the University of Cologne (16–404). Each participant gave written informed consent before study participation. The research was conducted in accordance with the Declaration of Helsinki.

3.1.1 Speakers, task and speech materials. We recorded 27 German monolingual native speakers with 3D Electromagnetic Articulography (EMA) using a Carstens AG501 articulograph and acoustically using a head-mounted condenser microphone. The EMA data itself is not reported in this paper as we only deal with the intonation here. The speakers were aged between 19 and 35 at the time of recording. 17 of them were female, 10 were male. No subjects with a special training in phonetics, phonology or prosody were included. None of the participants reported any speech or hearing impairments. The actual recording session after the participant had been prepared lasted about 45 minutes including a training session.

The participants were seated in front of a screen and were involved in an interactive game. They were told that in the game two robots work in a robot factory. One of them likes to hide the tools. The other one–a bit older and outdated in technology–needs help from the participant to retrieve those tools. In each trial, the participant saw the first robot placing the tool on an object in the factory room and leaving the scene. The second, older robot entered the scene but stopped in front of the closed factory door asking a question about the action of the first robot. Then, after the participant answered, the door opened, the second robot entered the room, took the tool and left the scene. The questions served as triggers for the focus structure of the answer and were chosen such that the target word denoting the object that the tool is placed on could be in *background*, *broad focus*, *narrow focus* or *contrastive focus*. Table 1 shows examples for such question-answer-pairs, the square brackets and the subscript F mark the focus domain. The question was given auditorily and in addition shown as a picture combination in a thought bubble above the head of the robot: the question tool on top of the question object in the case of background and contrastive focus; a simple question mark in the case of broad focus; the object and the question word "wo?" ("where?") with a question mark in the case of narrow focus. The answers were always given orthographically at the bottom of the screen, but many participants reported that they were able give the answers without reading after some trials.

As target words 20 German sounding disyllabic nonce words with a $C_1V_1{:}C_2ə$ structure were chosen. The words were designed to have stress on the first syllable and all participants pronounced them as expected. The first consonant was chosen from the set of /n m b l v/, the second consonant from /n m z l v/. The first, stressed vowel was either /a:/ or /o:/, the second always schwa. The consonants and vowels were combined such that each first consonant occurred twice with each first vowel and each second consonant-schwa-combination occurred

Table 1. Example question-answer-pairs to elicit the focus structures. The target word in these examples is "Wohse".

Focus structure	Example trigger and target sentence
Background	Q: Hat er die Säge auf die Wohse gelegt?<br>*Did he put the saw on the Wohse?*<br>A: Er hat [den Hammer]$_F$ auf die Wohse gelegt.<br>*He put the hammer on the Wohse.*
Broad focus	Q: Was hat er gemacht?<br>*What did he do?*<br>A: Er hat [den Hammer auf die Wohse gelegt.]$_F$<br>*He put the hammer on the Wohse.*
Narrow focus	Q: Wo hat er den Hammer hingelegt?<br>*Where did he put the hammer?*<br>A: Er hat den Hammer [auf die Wohse]$_F$ gelegt.<br>*He put the hammer on the Wohse.*
Contrastive focus	Q: Hat er den Hammer auf die Mahse gelegt?<br>*Did he put the hammer on the Mahse?*<br>A: Er hat den Hammer auf [die Wohse]$_F$ gelegt.<br>*He put the hammer on the Wohse.*

https://doi.org/10.1371/journal.pone.0216859.t001

four times in the whole set. Special care was taken that the words did not overlap with real German words. Nonce words with this specific constant structure were chosen since the study was also designed to measure articulator movements using EMA. All words were given with the female determiner “die”. The target words are given in S1 Table.

Each target word was associated with a fictitious visual object. The participants were presented with all tools and target words in a preparation phase before the training phase and the experiment and were asked to read the words aloud with the determiner “die” (“die Nohme”, “die Lahse”, etc.).

In the experimental game, tools are placed on the objects. Each object was hence paired with a tool to occur with. The tools are given in S2 Table. As there are 10 tools and 20 target words, each tool had to occur twice. Furthermore, for the background condition and the contrastive condition a competitor tool or object was needed respectively (for the direct object of the question when the target word was in background: “Did he place **X** on A?” “He placed Y on A!”; and for the indirect object of the question when the target word was in contrastive focus: “Did he place X on **A**?” “He placed X on B!”). These combinations were fixed for each participant, yielding 20 quadruples of target object, tool, competitor object and competitor tool. The competitor object was chosen such that the first consonant or the first vowel did not equal the first vowel or consonant of the target object. The competitor tool was chosen such that it differed in the first consonant from the target sentence tool. The 20 quadruples occurred with all four focus conditions, which resulted in a total of 80 trials. 16 training trials with different object-tool-quadruples preceded the actual experiment session.

The randomisation of the trial order was done using a Python script. Subsequent trials were not allowed to contain the same target word or tool used in the target sentence. Furthermore, no three subsequent trials with the same focus condition were allowed. For two subsequent trials with identical focus condition an upper limit was set: In only 15% of the list, two adjacent trials with equal focus conditions were possible. All 27 participants received different randomised lists of trials.

The scenes, objects, tools, and robots were drawn by a professional book illustrator. The game was developed as an interactive website using HTML and JavaScript with jQuery to animate the robots’ movements (including arm and mouth movement) and the door movement. The experimenter, sitting behind the participant, pressed a key on the keyboard to make the robot move towards the tool and proceed to the next trial. There was a “rescue key” to repeat the trial in case something went wrong. Between trials, the scenery disappeared and the participant saw areas of changing light colours for four seconds. This was done to detach the trials from one another to make sure that the focus structure of the target sentence made reference to the current trial only. Points were counted in the lower right corner of the screen to make the task more game-like. Fig 7 shows an example of the experiment screen, where the second robot has just asked its question and is waiting for the answer. The code of the experiment app is available for download: DOI: 10.5281/zenodo.2611287 (http://doi.org/10.5281/zenodo.2611287).

3.1.2 Measures. We measured the *tonal onglide* of each nuclear pitch accent. The tonal onglide describes the f0 movement towards the main target of the pitch accent [52]. In autosegmental-metrical phonology, a model of intonation in which pitch accents are represented as tones associated with stressed syllables [53], a L+H* or H* pitch accent is characterised by a rising movement up to the target of the H* whereas a H+L* or H+!H* pitch accent is characterised by a falling movement down to the L* or !H*. The tonal onglide captures both the direction (“rising or falling?”) as well as the magnitude of the movement (“how much is it rising or falling?”). Negative onglide values represent accents that are characterised by an early high f0 (either in the syllable before or at the beginning of the accented syllable) and a falling movement towards or throughout the accented syllable. Positive onglide values, on the other

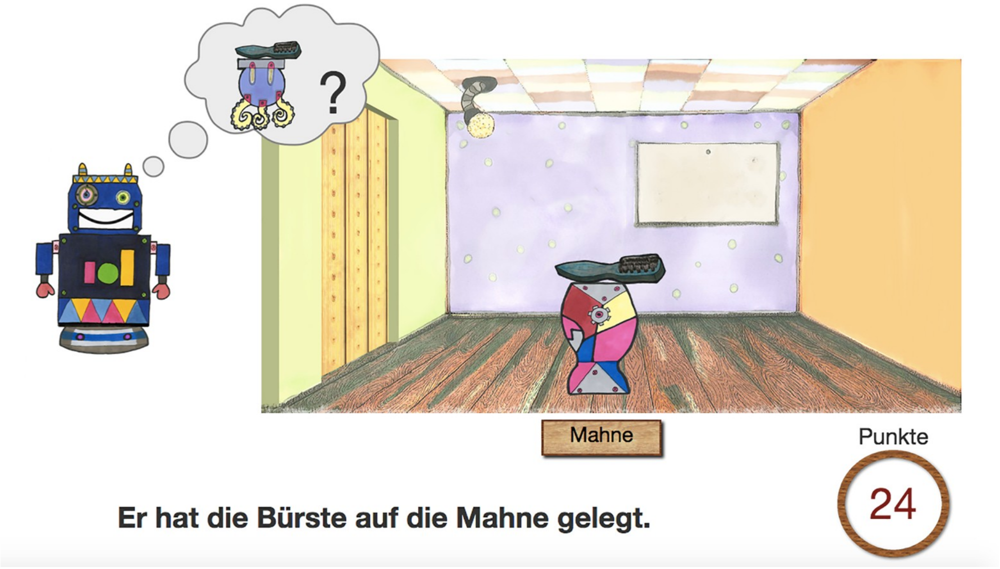

Fig 7. Example screen from the experiment during a trial with contrastive focus condition. (Image by Isabel Schwegel and Simon Roessig, CC-BY 4.0).

https://doi.org/10.1371/journal.pone.0216859.g007

hand, represent accents with a lower f0 at the beginning and a rise within the accented syllable towards a high f0 target.

Two labellers with a training in prosody worked together on a consensus annotation of the data in Praat [54]. The labellers did not know the intended focus structure of the sentence they worked on. In the rare cases of disagreement, the labellers discussed the contour until they found a consensus transcription. In all cases, the labellers were able to find a solution rapidly. First, all utterances were identified in which the speaker did not place the nuclear pitch accent on the target word. This group of utterances comprised more than 99% of background condition productions as well as a minority of productions from the other conditions. Since these tokens do not have a nuclear pitch accent on the target word, it is not possible to do an onglide analysis. Because only less than 1% of the utterances in background condition exhibited the nuclear accent on the target word, we decided to exclude this condition completely for the purpose of this study. It should be noted that the deaccentuation of background targets was expected. Second, the labellers judged perceptually whether the nuclear pitch accent was falling or rising. Third, the beginning and the end of the onglide movement were manually identified within a three-syllable-window including the accented syllable, the syllable before and the syllable after. Fig 8 gives a schematic depiction of the onglide measure for both rising and falling accents.

In the case of rising accents, a local minimum just before the rising movement was labelled in the pre-accented syllable or the accented syllable itself as the beginning of the onglide movement. A local maximum right at the end of the rise was labelled in the accented syllable or the post-accented syllable as the end of the movement.

In the case of falling accents, a relatively high point right before the falling movement was labelled in the pre-accented syllable or the accented syllable itself as the beginning of the onglide movement. Since the pitch is usually falling through the syllable in a falling accent and hence a pitch target is virtually impossible to determine, the middle of the vowel was marked as the end of the accentual movement.

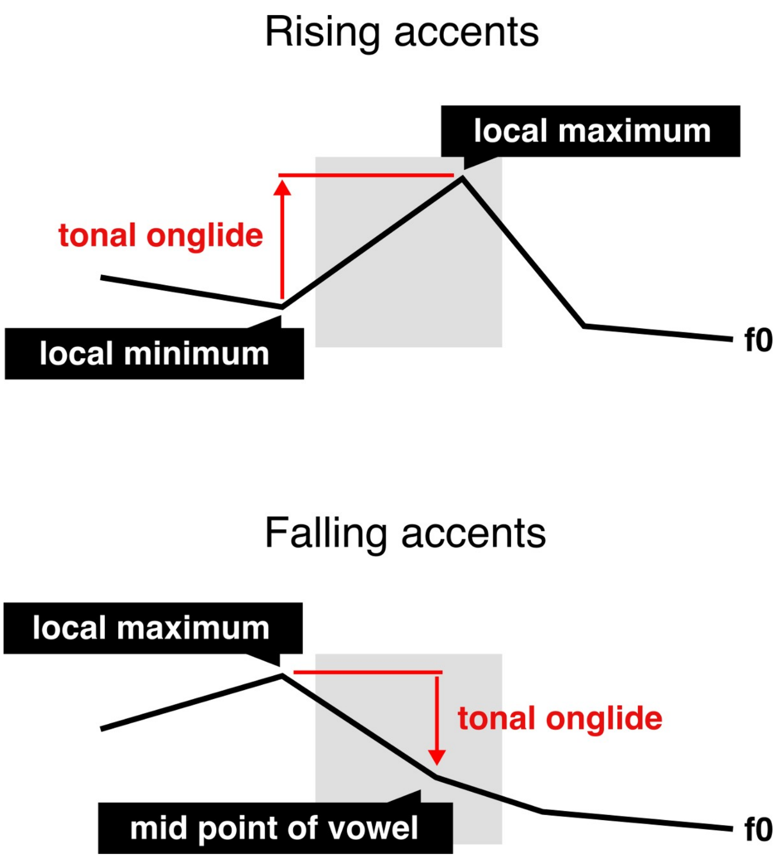

Fig 8. Schematic depiction of the onglide measure for rising accents (top) and falling accents (bottom).

https://doi.org/10.1371/journal.pone.0216859.g008

To avoid the influence of micro-prosodic effects, labels for low points (i.e. the beginnings of rises) were exclusively labelled in vowels. Labels for high points (i.e. the beginnings of falls and the ends of rises) were placed in voiced consonants only in some clear cases. This procedure is conservative in so far as voiced consonants are generally said to lower the f0 [19]. Thus, putting high targets in voiced consonants can only lead to less extreme, more centralised onglide values. In addition, the labellers avoided placing f0 labels in the first milliseconds after the release of a stop, as this region is known to be affected by micro-prosodic influences of consonants as well [55]. Onglide measures were extracted using Praat and the difference in semitones between the beginning and the end of the onglide movement was calculated. The data set of onglide values as well as the software used to analyse the data is available online: DOI: 10.5281/zenodo.2611316 (https://doi.org/10.5281/zenodo.2611316).

In addition to the exclusion of those tokens without a nuclear pitch accent on the target word, a minority of cases had to be excluded due to mispronunciations or strong disfluencies. All in all, the data set comprised 1554 onglide values. This number seems rather low given that 80 utterances were recorded from 27 speakers each (27 speakers x 4 conditions x 20 targets = 27 x 80 = 2160). However, the nuclear pitch accent placement on the subject in the background condition was anticipated in the design. Thus, only 60 utterances of each speaker (3 conditions x 20 targets) were expected to bear a nuclear pitch accent on the target word, i.e. 1620 in total (27 speakers x 3 conditions x 20 conditions).

3.2 Results

Fig 9 shows the onglide distributions for all speakers together for each of the three focus conditions as violin plots. In the plots, it is already possible to see the differences in the distributions: Broad focus seems to have a bimodal distribution with strong modes on both sides of zero, with almost equal numbers of rises and falls; narrow focus has a clearly asymmetrical bimodal distribution with more rises than falls; contrastive focus has even fewer falling pitch accents and almost exclusively rising pitch accents.

A closer inspection of the data reveals that the speakers differ quite substantially in their relative pitch ranges. While some speakers talk in a lively manner, others are rather subdued. While an onglide value of +5.5 semitones might be quite extreme for a speaker with a mean of +3 semitones for rising onglides, the same value might not be extreme for a speaker with a mean of +5 semitones for this type of accent. This leads to a substantial skew in the data. Because speakers are going to be analysed together here with a dynamical model that produces near normal distributions as outputs and because the exact values of the individual speakers are not the main interest, we can use a log transformed scale here. [The data (denoted here by Y) is transformed in the form sign(Y) * log(abs(Y+1)), i.e. the absolute values are log transformed after adding a constant, the result of the transformation is then multiplied with −1 or +1 to retain the original sign of the value. The constant has to be added because log yields negative results for values greater than 0 and smaller than 1. Using the absolute value and restoring the sign after the transformation has to be carried out because log is only defined for positive values. Values of 0 did not exist in the data set.] Fig 10 gives the results after the normalisation. The main pattern in the data is preserved with the normalisation, but the symmetry of the distribution in broad focus comes out even clearer now. Table 2 gives the raw counts and proportions of falls and rises for each focus condition.

Besides the differences in proportions of falling and rising onglides, one can observe in the violin plots that the distributions shift slightly to the right when going from broad to narrow, and finally to contrastive focus. This means that the rising onglides become increasingly high.

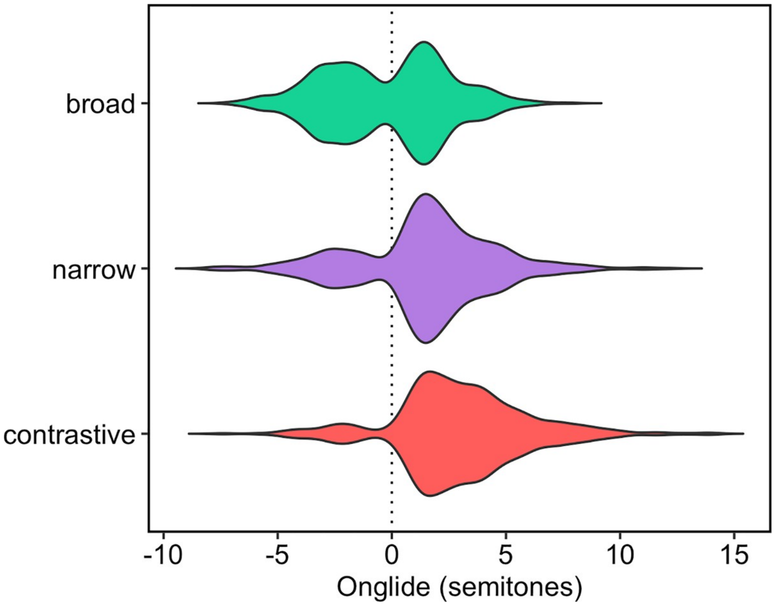

Fig 9. Distributions of raw onglide values pooled across all speakers in semitones. Negative values indicate falling accents, positive values indicate rising accents.

https://doi.org/10.1371/journal.pone.0216859.g009

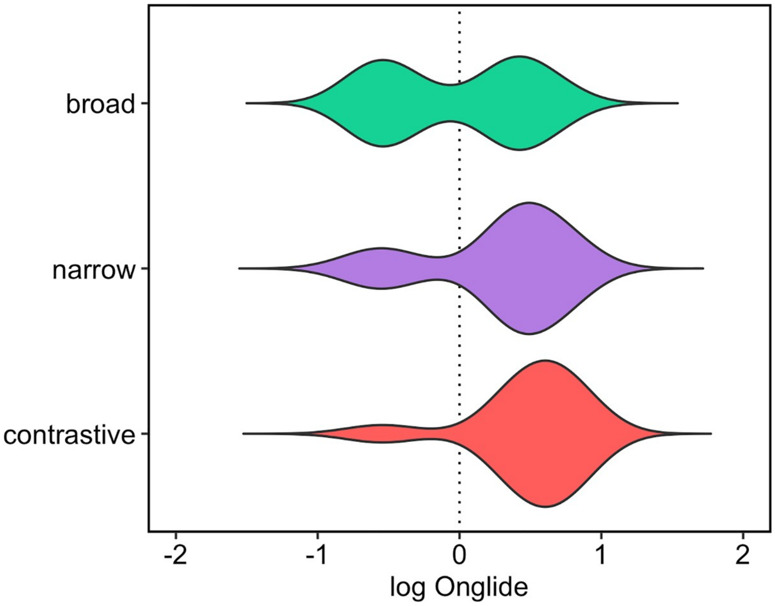

Fig 10. Distributions of normalised onglide values pooled across all speakers.

https://doi.org/10.1371/journal.pone.0216859.g010

This phonetic variation happens in addition to the difference in the distribution of falling and rising pitch accents. To assess the shift of the distributions numerically, we calculated the median of the *rising* onglides, i.e. we took only the distributions of values higher than zero. Fig 11 presents the results. A step-wise increase of the median can be observed from broad to narrow, and from narrow to contrastive focus.

We analysed the results using a Bayesian linear mixed model in R [56] with brms [57] that implements an interface to Bayesian inference in Stan [58]. We report the estimated differences between focus conditions in terms of posterior means, 95% credible intervals, and the probability of the estimate being greater than 0. Given the data and the model, the 95% credible intervals indicate the range in which one can be certain with a probability of 0.95 that the difference between estimates can be found (note that these probabilities are not p-values). To calculate the differences between focus types, we subtracted the posterior samples for broad focus from narrow focus (narrow–broad), narrow focus from contrastive focus (contrastive–narrow), and broad focus from contrastive focus (contrastive–broad).

The model includes the log transformed onglide as the dependent variable, focus type as a fixed effect, and random intercepts for speakers and target words as well as by-speaker slopes for the effect of focus type. As the distribution of the dependent variable is bimodal, we use a prior for the predictor that is characterised by a mixture of two gaussian distributions centred around -0.5 and 0.5 respectively. The model estimates the parameter theta that represents the extent to which the two gaussian distributions are mixed. For this parameter, we use a prior centred around 0. Differences in theta indicate the differences in the proportions of the two

Table 2. Numbers and proportions of falls and rises in the three focus types.

Focus Type	Falls	Rises
Broad	240 (47.0%)	271 (53.0%)
Narrow	115 (21.9%)	411 (78.1%)
Contrastive	47 (9.1%)	470 (90.9%)

https://doi.org/10.1371/journal.pone.0216859.t002

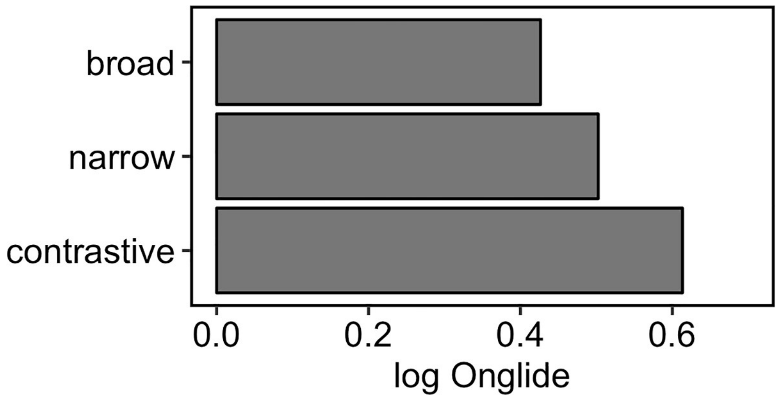

Fig 11. Medians of the rising normalised onglide distributions for the three focus types pooled across all speakers.

https://doi.org/10.1371/journal.pone.0216859.g011

modes in the onglide data. The model runs with four sampling chains of 3000 iterations each, with a warm-up period of 1500 iterations.

Given the model and the data, the analysis yields differences in the posterior probabilities for the mixing parameter theta between broad focus and narrow focus ($\hat{\beta} = 1.33, 95\%$ $CI = [0.64, 1.94], \Pr(\hat{\beta} > 0) = 1$), narrow focus and contrastive focus ($\hat{\beta} = 1.61, 95\%$ $CI = [0.52, 2.62], \Pr(\hat{\beta} > 0) = 1$), as well as broad focus and contrastive focus ($\hat{\beta} = 2.94$, 95% $CI = [1.65, 4.16], \Pr(\hat{\beta} > 0) = 1$). In all cases, the estimated differences are positive indicating a growth of the right mode from broad to narrow focus, from narrow to contrastive focus, and from broad to contrastive focus.

To assess the differences between the focus conditions regarding the rising distributions, we investigate the mean estimates of the right gaussian sub-distribution. There are differences in the posterior probabilities between broad focus and narrow focus ($\hat{\beta} = 0.06, 95\%$ $CI = [0.04, 0.09], \Pr(\hat{\beta} > 0) = 1$) narrow focus and contrastive focus ($\hat{\beta} = 0.08, 95\%$ $CI = [0.05, 0.11], \Pr(\hat{\beta} > 0) = 1$) as well as broad focus and contrastive focus ($\hat{\beta} = 0.15, 95\%$ $CI = [0.11, 0.18], \Pr(\hat{\beta} > 0) = 1$). In all cases, the estimated differences are positive indicating that the model estimates the rises to become increasingly large from broad focus to narrow focus, from narrow focus to contrastive focus, and from broad focus to contrastive focus.

In the next section, we turn to a dynamical systems approach that accounts for the changes in the proportions of the categories (rising and falling) and the phonetic realisation of the rises (increase in magnitude of rises).

3.3 A modelling account

For the modelling, we use a dynamical system with two attractors, assuming two types of pitch accents: falling and rising. However, this is not to say that there are only two nuclear pitch accent types in German in general. The two pitch accent types can be represented by an attractor landscape with two stable states, one for falling and one for rising accents–similar to the one shown in section 2 and used by [5,6] and [36] among others. The x variable of our system represents the tonal onglide. Remember that a negative onglide value indicates a falling accent, while a positive onglide value indicates a rising accent. The system chosen here to model an attractor landscape for falling and rising pitch accents is given by the potential energy function in (5) and the force function in (6) (the constant model parameters were chosen by trying out different pairs of values such that the model is able to simulate data in the

range of the measured data–they may not be optimal but the correspondence to the real data is quite good as will become clear later). Fig 12 shows a graph of the potential energy V(x) and force F(x) when k = 0. It is clear to see that the system's potential energy has a symmetrical shape with two identical valleys on the left and the right side of the zero-line. Thus, for k = 0, the system's state can equally gravitate towards falling or rising pitch accents.

$$V(x) = 18x^4 - kx^3 - 7.5x^2 \tag{5}$$

$$F(x) = -(72x^3 - 3kx^2 - 15x) \tag{6}$$

As shown in the introductory section on dynamical systems (section 2), the attractor landscape can be altered by scaling the control parameter k. It can be tilted to the left or the right side to gradually increase or decrease the strength of one of the attractors. The outcome of the system for different values of k can be tested via simulation. The simulation lets us evaluate the predictions of the system and gain insights into how well the model can project the real data. We use a simulation method that is inspired by the software accompanying [5], the code is implemented in R with parts in external C++ code. The source code is available for download: DOI: 10.5281/zenodo.2611316 (https://doi.org/10.5281/zenodo.2611316).

The simulation operates on the force function formulated in (6) as the negative derivative of the potential energy function and shown in Fig 12 (right panel). It starts at a randomly chosen state and iterates in small time steps over the function using a stochastic version of the Euler method [59]. This method calculates the change of the system at the current state x and adds it to that state to get to the next state, just like shown in the introductory example above with the parabola shaped potential energy function. After a fixed period of time, i.e. a fixed number of small time steps, a single simulation run stops and the current state is registered as the result. This procedure reflects the fact that cognitive processes unfold in time [12,47,60] but the time window is not indefinite, in reality it is probably extremely short. For the sake of simplicity, the simulation implements a time window that always has the same length. The step size is chosen sufficiently small for the Euler method to produce accurate results. Crucially, during each step of the simulation, Gaussian noise from a normal distribution with a mean of 0 and a standard deviation of 1 is added to the result since noise is a ubiquitous component of complex systems [5,51]. By adding noise, the simulation results represent the

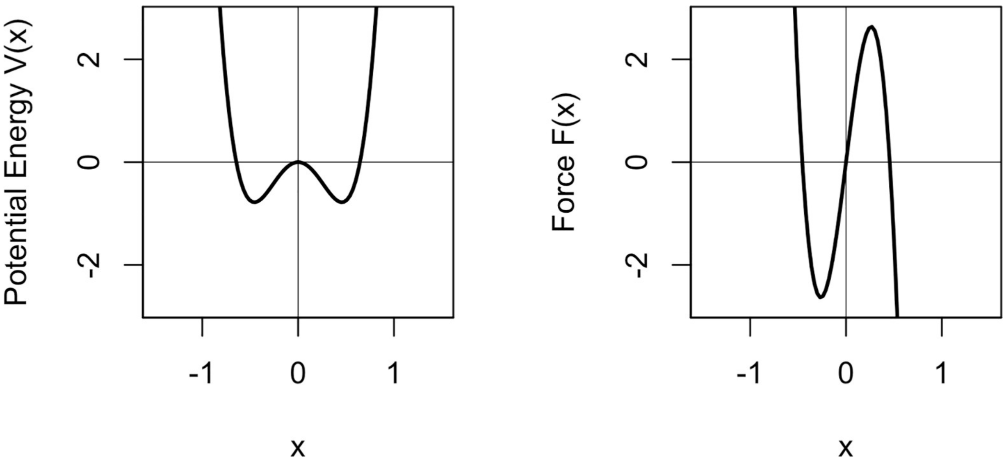

Fig 12. Potential energy function V(x) from (5) and force function F(x) from (6) for k = 0.

https://doi.org/10.1371/journal.pone.0216859.g012

stability of the attractor: Noise pushes the system away from its current state. The more stable an attractor is, the smaller the influence of noise on this state. In other words, when the system is close to a stable attractor, the probability is high that it will stay in the basin of the attractor despite the noise. On the contrary, when the system is near a less stable attractor, it is more likely to be pushed away from the attractor's basin–eventually ending up in the vicinity of the more stable attractor. The simulation is run for 2500 data points and with a random initial state in each of the 2500 simulation runs. A single simulation run corresponds to a speaker producing one nuclear pitch accent.

Fig 13 shows how the simulation results will look for different values of the control parameter k. The figure shows the simulation data for three different, rather extreme values of k: -6, 0, and 6. The three panels on the top show the attractor landscapes in terms of potential energy functions. The bottom panel gives the simulation results in violin plots for these three k values. It can be seen how the system develops from being tilted to the left to being tilted to the right when k is increased. When k is negative, the left attractor is more stable, thus the system will settle most often in this attractor basin, yielding most of the data in this region. Conversely, when k is positive, the right attractor is more stable and the system will settle in this attractor basin most often. Interpreted as a hypothetical system for intonation, with k = -6 the system will produce almost only falling pitch accents, with k = 6 it will produce almost only rising pitch accents, while the two pitch accent types are equally likely for k = 0.

Note that we have chosen values for k with rather extreme distances in this example. More subtle changes of the control parameter will result in more fine-grained differences of the system's outcome: Fig 14 shows potential energy functions and simulation results for k = 3 and k = 6. The horizontal lines show the medians of the positive portions of the distributions, dashed and red for k = 3, solid and blue for k = 6. Although the distributions for the two k values look quite similar having predominantly values on the right side, we can observe two important differences of the system's outcome here: First, for k = 3 the simulation produces a slightly higher proportion of negative values, i.e. the system settles more often in the left attractor. Second, the medians of the positive portions of the distributions (only values higher than 0) are different with the results for k = 6 having a higher median than the results for k = 3. The two examples illustrate how our dynamical system can reproduce two important aspects of the intonation data presented in the results section: changes in the proportions between two categories and more subtle shifts in the continuous distribution within these categories.

Having set up the model and the simulation method, the question arises which values for the control parameter k best model the data of the three different focus types. The question can be answered by calculating how much the distributions of the real data and the simulated data for a given k differ. The smaller the distance, the better the value for the modelling. We have shown that the distributions of the focus types differ in two respects: (1) the balance of the distribution, quantified by the ratio of counts of rising and falling, and (2) the location of the rising (or positive) portion of the distribution, quantified by the distribution median. As shown above in the two simulation examples, the model is able to capture both of these aspects. Hence, these criteria are used to calculate the distance between the real data under scrutiny and the simulated data.

We use this method to iteratively find the most plausible k value. For each possible k in a range between -5 and 10 with a step size of 0.1, data is simulated with the model. Ten simulations are run for each k and, as stated above, each simulation run results in 2500 data points. The simulated data for all potential k values within the interval are tested against the real data. For each potential k, the two distances corresponding to the two criteria outlined above are calculated: (1) the absolute difference of the ratios of counts of falling and rising accents for the real data and the simulated data; (2) the absolute distance of the medians of the rising accents

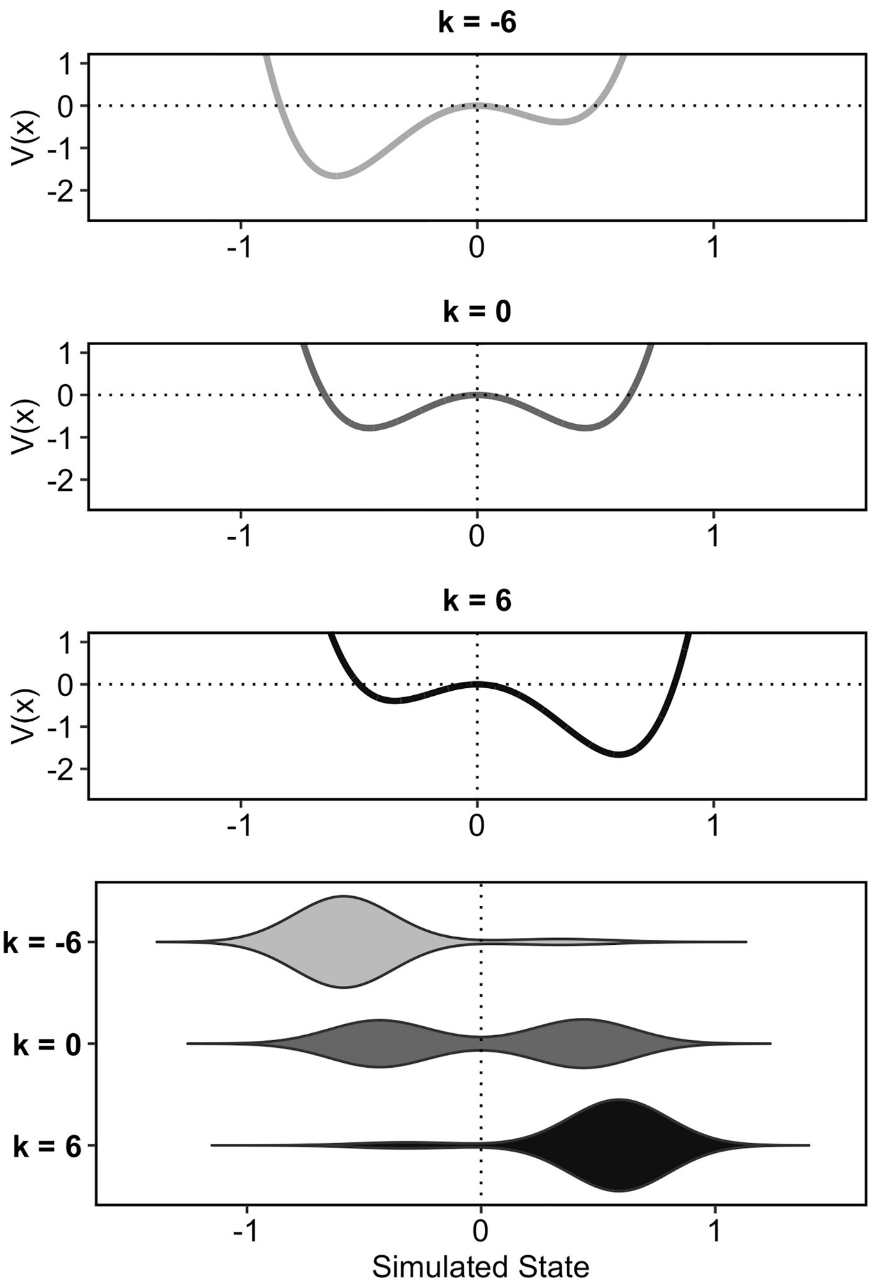

Fig 13. Illustration of the simulation with different values for k. The first three panels from the top show the potentials for different values of the control parameter k. The lower panel gives the simulation results for these parameter values.

https://doi.org/10.1371/journal.pone.0216859.g013

of the real data and the simulated data. Since 10 simulation runs are performed for each k, the means for both the ratios and the medians of the rising distribution have to be calculated for the data of the 10 simulations for comparison. This is done to achieve a robust estimate of the simulated distributions, since the simulation includes random noise. For each potential value of k, the absolute distances of the two sets are added for each k respectively. The k value associated with the minimum of these added distances is chosen as the winning candidate. S1 Fig shows the added distances for each tested k for all focus types.

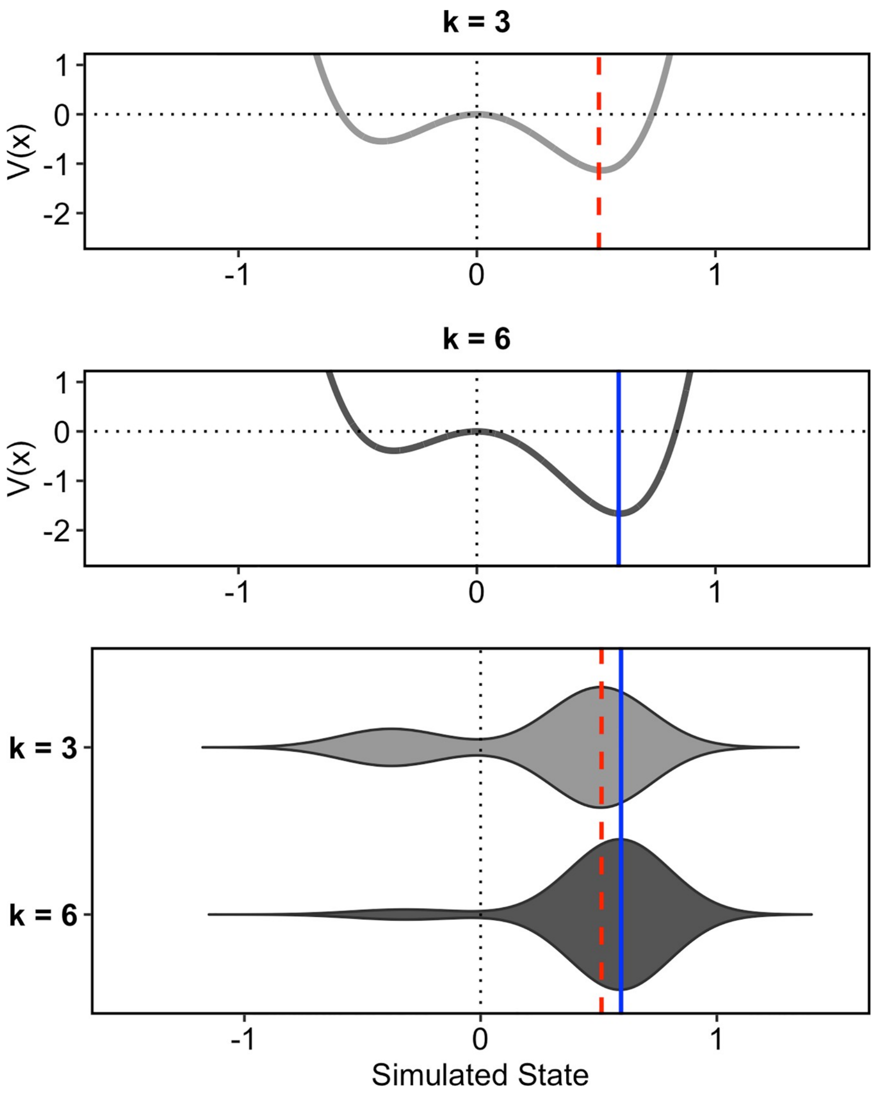

Fig 14. Another illustration of the simulation with different values for k. The first two panels from the top show the potentials for different values of the control parameter k. The lower panel gives the simulation results for these parameter values. The horizontal lines give the medians of the positive portions of the distributions, dashed/red for k = 3, solid/blue for k = 6.

https://doi.org/10.1371/journal.pone.0216859.g014

Fig 15 shows the results of the simulation using the outlined method. The attractor landscape described by the energy curve is given, along with the simulation results. It turns out that broad focus accents can overall be best modelled with k = 0.3 while narrow focus accents are best modelled with k = 3.3 and contrastive focus accents are best modelled with k = 5.4. Comparing the results to the violin plots of the real data (Fig 10), it can be observed how the simulation captures the change in the proportions of rising and falling onglides by tilting the attractor landscape to the right with higher k values and thereby stabilising the right attractor. The increase in the control parameter also results in higher distribution medians like shown in Fig 16. This pattern resembles the pattern found in the medians of the real data, cf. Fig 9.

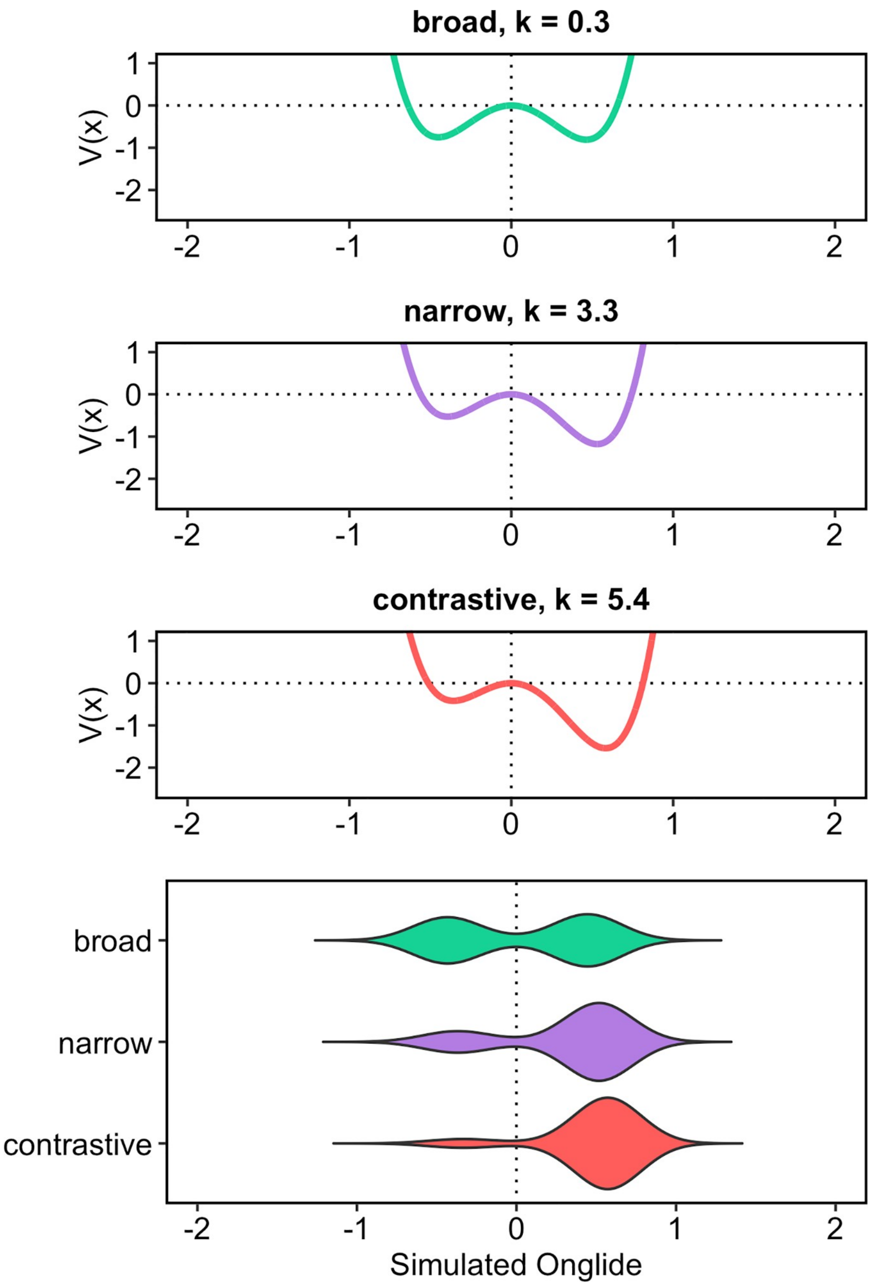

Fig 15. Attractor landscapes and simulated data for broad, narrow and contrastive focus with the best fitting values for the control parameter k. The first three panels from the top show the potentials for the three focus types. The lower panel gives the simulation results.

https://doi.org/10.1371/journal.pone.0216859.g015

3.4 Modelling speaker groups

With the dynamical model for pitch accents established, the question arises as to whether all speakers use the mechanisms of the system in the same way. Even with as few subjects as five, [35] could observe different strategies among the speakers: One group used qualitative variation–i.e. falling vs. rising–to differentiate between focus types. The other group used rising pitch accents only but produced more subtle quantitative variation in the magnitude of rising onglides,

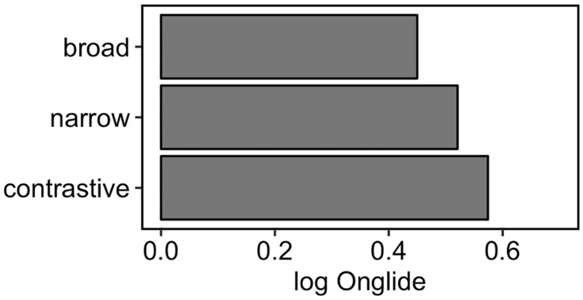

Fig 16. Medians of the rising simulated onglide distributions for the three focus types.

https://doi.org/10.1371/journal.pone.0216859.g016

with narrow focus having larger onglides than broad focus, and contrastive focus having larger onglides than narrow focus. To assess these differences in the present data set, we grouped speakers according to their overall pattern of pitch accent productions. Group 1 consists of the 11 speakers who use falling onglides in more than 33% of the cases overall. Group 2 consists of the 16 speakers who use up to 33% falling onglides overall. Note that, in principle, it would have been possible to model each speaker separately. However, a single speaker contributes a maximum of only 20 utterances per focus condition (utterances were sometimes missing due to technical problems or mispronunciations). As the calculation of k values proves to be far more reliable with more data points, this more coarse-grained division into groups was preferred.

In Fig 17, the log-transformed onglide distributions of the two groups are shown: left for group 1 and right for group 2. For group 1, the distributions of broad, narrow and contrastive focus are more distinct: In broad focus, falling accents are most frequent; in narrow focus the distribution of falling and rising onglides is almost symmetrical; in contrastive focus there is only a small number of falling accents. For group 2 the distributions are less distinct: Rising onglides are predominantly used in all three focus types, although there is a small number of falling accents in broad focus and an even smaller number of falling accents in narrow focus.

Fig 18 presents the medians of the rising onglide distributions for the two speaker groups. For both groups, the medians increase when going from broad through narrow to contrastive focus, indicating that rising onglides become higher. In addition to this main trend, the medians for group 2 are higher overall than those of group 1.

We repeated the same Bayesian analysis as before for each speaker group separately. The structure of the model remained constant. For group 1, given the model and the data, the analysis shows that there are clear differences in the posterior probabilities for the mixing

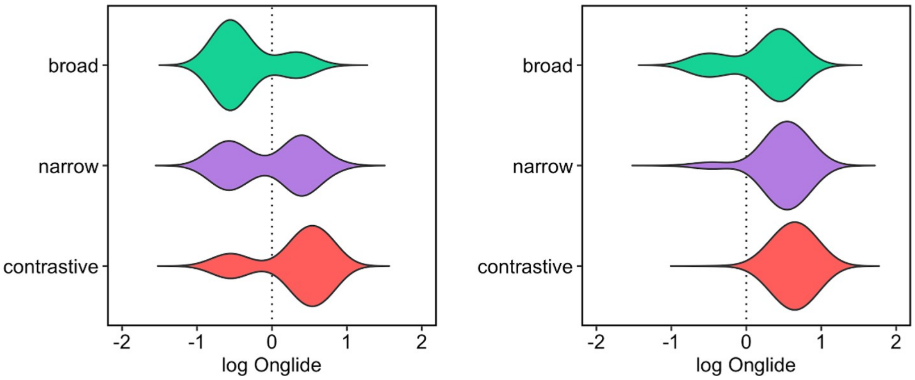

Fig 17. Onglide distributions of group 1 (left) and group 2 (right).

https://doi.org/10.1371/journal.pone.0216859.g017

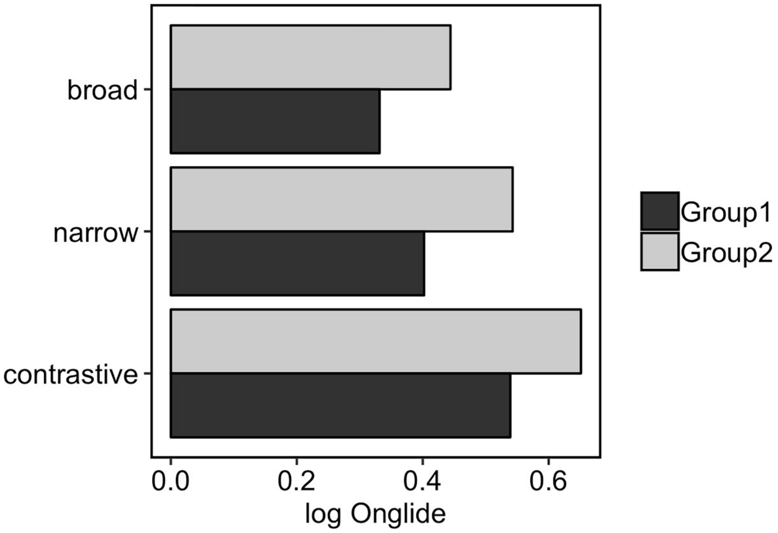

Fig 18. Medians of rising onglide distributions for the two speaker groups.

https://doi.org/10.1371/journal.pone.0216859.g018

parameter theta between broad focus and narrow focus ($\hat{\beta} = 0.95, 95\%\ CI = [-0.01, 1.89]$, $\Pr(\hat{\beta} > 0) = 0.96$), as well as broad focus and contrastive focus ($\hat{\beta} = 1.51, 95\%\ CI = [-0.27, 3.25]$, $\Pr(\hat{\beta} > 0) = 0.94$). The model also yields evidence for a difference in the mixing parameter between narrow focus and contrastive focus but this evidence is weaker with a probability of 0.76 ($\hat{\beta} = 0.56, 95\%\ CI = [-1.14, 2.12]$, $\Pr(\hat{\beta} > 0) = 0.76$). All in all, the analysis shows that the mixing parameter is different between the focus types with the right mode growing from broad to contrastive. Narrow seems to be closer to contrastive but still lies in between the two focus types.

Regarding the estimates of the rising sub-distribution, the model provides evidence for differences in the posterior probabilities between broad focus and narrow focus ($\hat{\beta} = 0.05$, 95% $CI = [-0.01, 0.11]$, $\Pr(\hat{\beta} > 0) = 0.95$) narrow focus and contrastive focus ($\hat{\beta} = 0.07$, 95% $CI = [0.01, 0.14]$, $\Pr(\hat{\beta} > 0) = 0.98$) as well as broad focus and contrastive focus ($\hat{\beta} = 0.12$, 95% $CI = [0.06, 0.19]$, $\Pr(\hat{\beta} > 0) = 1$). In all cases, the estimated differences are positive indicating that the model estimates the rises to become increasingly large from broad focus to narrow focus, from narrow focus to contrastive focus, and from broad focus to contrastive focus for group 1.

For group 2, given the model and the data, there is evidence for differences in the posterior probabilities for the mixing parameter theta between broad focus and narrow focus ($\hat{\beta} = 1.13, 95\%\ CI = [0.01, 2.14]$, $\Pr(\hat{\beta} > 0) = 0.97$), narrow focus and contrastive focus ($\hat{\beta} = 1.31, 95\%\ CI = [-0.38, 2.80]$, $\Pr(\hat{\beta} > 0) = 0.94$), as well as broad focus and contrastive focus ($\hat{\beta} = 2.44, 95\%\ CI = [0.70, 4.00]$, $\Pr(\hat{\beta} > 0) = 0.99$). In all cases, the estimated differences are positive indicating a growth of the right mode from broad to narrow focus, from narrow to contrastive focus, and from broad focus to contrastive focus for group 2.

Regarding the estimates of the rising sub-distribution, the model shows differences in the posterior probabilities between broad focus and narrow focus ($\hat{\beta} = 0.07, 95\%\ CI = [0.04, 0.10]$, $\Pr(\hat{\beta} > 0) = 1$) narrow focus and contrastive focus ($\hat{\beta} = 0.08, 95\%\ CI = [0.04, 0.12]$, $\Pr(\hat{\beta} > 0) = 1$) as well as broad focus and contrastive focus ($\hat{\beta} = 0.15, 95\%\ CI =$

$[0.11, 0.19], \Pr(\hat{\beta} > 0) = 1)$. In all cases, the estimated differences are positive indicating that the model estimates the rises to become increasingly large from broad focus to narrow focus, from narrow focus to contrastive focus, and from broad focus to contrastive focus for group 2.

We used the same method to find the appropriate k values for the focus data of the two groups. In S1 Fig, the distances between the real data and the simulation results are shown for each potential k for both groups. The simulated onglide distributions and dynamical system potential energy curves are shown in Fig 19. The left panels show the potentials and simulated data for group 1, the right panels show the same for group 2. The results indicate that group 1 can be best modelled with k = –3.4 for broad, k = 0.5 for narrow and k = 3.4 for contrastive focus. Group 2 can be best modelled with k = 3 for broad, k = 6.2 for narrow and k = 8.4 for contrastive focus. Fig 20 provides the medians of the rising portions of the simulated data. The general trend of increasing medians with increasing k values from broad through narrow to contrastive focus can be observed in the simulated data and is consistent with the observed data, although the steps between the focus types appear slightly larger in the real data compared to the model for both groups. As to the relation between the two groups, our simulation

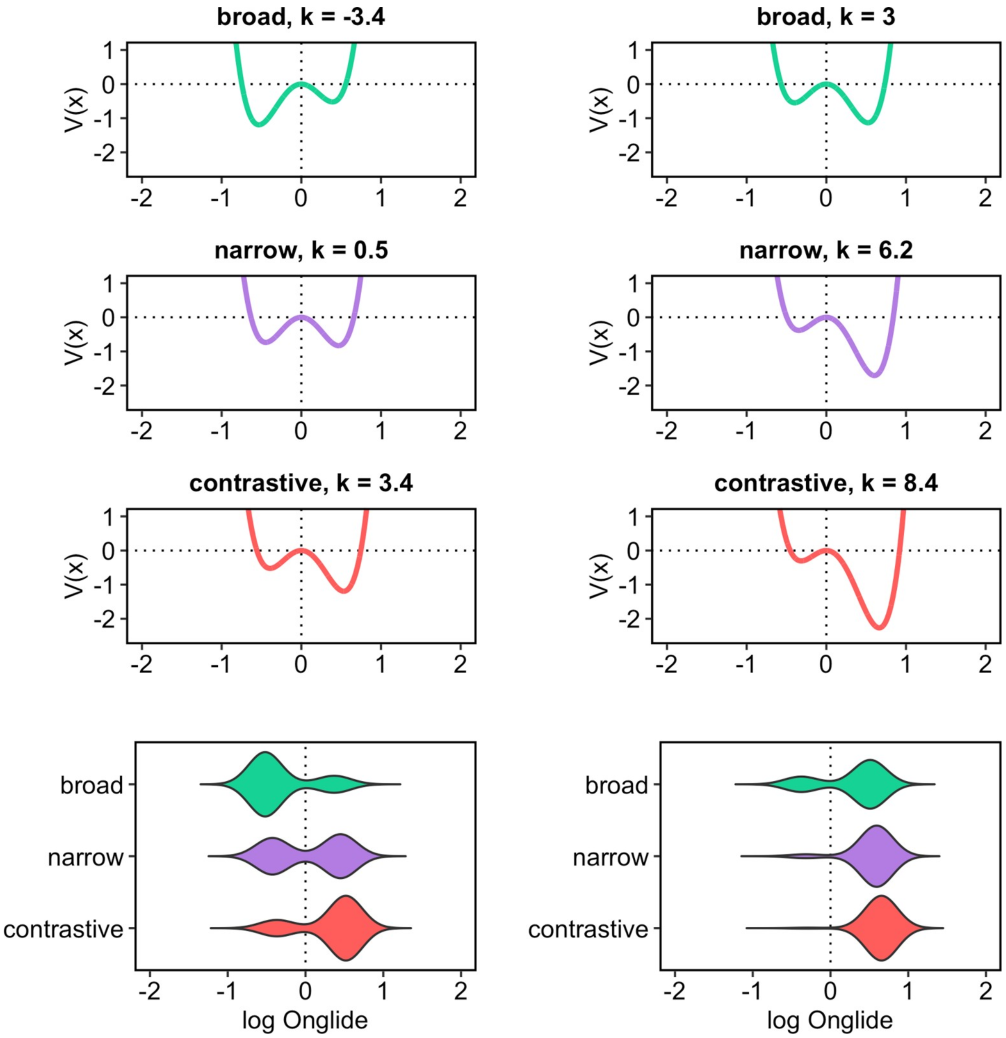

Fig 19. Attractor landscapes and simulated data for broad, narrow and contrastive focus with the best fitting values for the control parameter k. Group 1 on the left, group 2 on the right. The first three panels from the top show the potentials for the three focus types. The lower panel gives the simulation results.

https://doi.org/10.1371/journal.pone.0216859.g019

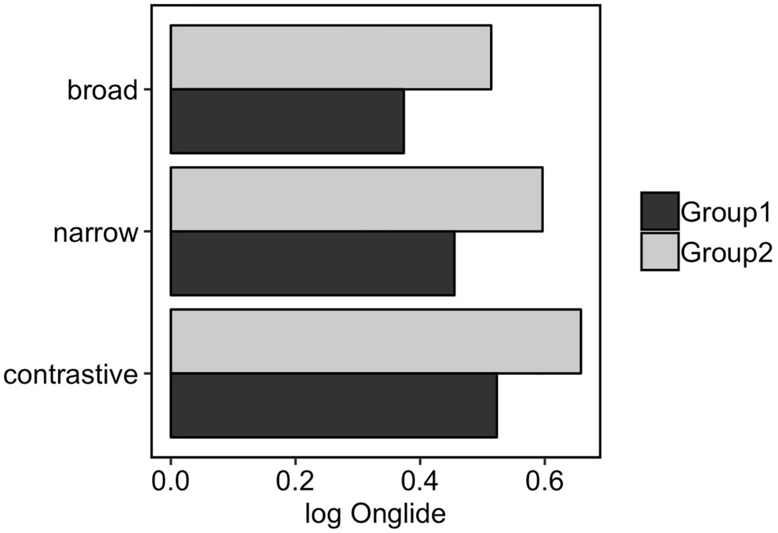

Fig 20. Medians of the rising simulated onglide distributions for all focus types and groups separately.

https://doi.org/10.1371/journal.pone.0216859.g020

is able to capture that the medians for group 2 are higher overall compared to the medians of group 1.

In this section, we presented the results from a production study with 27 speakers and a dynamical model that is able to capture the pitch accent patterns found for the productions of different focus types. The model can account for the qualitative aspects (falling vs. rising) as well as the quantitative aspects (less rising vs. more rising) in one system by means of scaling a single parameter. Crucially, it is also able to model group-specific strategies as differences in the scaling of this parameter.

Fig 21 reproduces the k values for both groups graphically to illustrate the differences and similarities. Speakers appear to use the potential space of values for this parameter differently: Group 1 starts low for broad, while group 2 starts high and goes even higher for narrow and contrastive focus than the other group. Crucially, however, k is always scaled in the same direction, increasing from broad to narrow and from narrow to contrastive focus. Metaphorically, one could say that speakers turn up the volume of the prosodic system to enhance prominence–a mechanism that results in more prominent pitch accent types when the focus domain is narrowed (broad to narrow) and when immediate contrast is introduced in the pragmatic

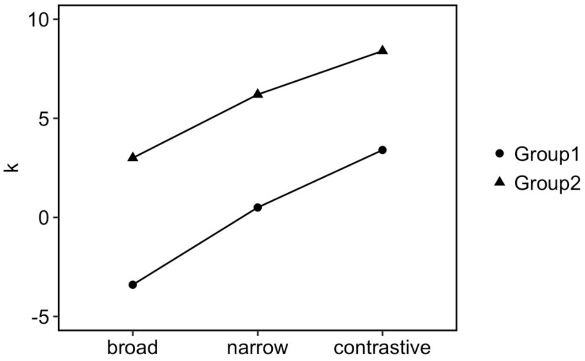

Fig 21. Control parameter values (k) of the two speaker groups.

https://doi.org/10.1371/journal.pone.0216859.g021

context (narrow to contrastive). But speakers behave differently when they adjust the volume for their prosodic realisations. Because the control parameter is the input to a non-linear dynamical system with two stable states, the volume control can result in both qualitative, phonological as well as quantitative, phonetic changes.

4. General discussion

Assuming phonology and phonetics operate at one level, we can use attractors to model both phonological categories and their phonetic implementation. The basic attractor landscape, in our model expressed by the equation V(x) in (5) when the control parameter k is 0, can be considered the grammar or phonology. In a given communicative context, a speaker scales the system appropriately to express a particular meaning (in this case, focus type). In our model this is achieved by the speaker selecting the appropriate value for the control parameter. We have also demonstrated that different groups of speakers use the scaling of the system differently. The following speaker group specific patterns can be captured with a single model by using different parameter value spaces:

i. The model is able to capture the behaviour of speakers who make a categorical distinction (falling vs. rising) to signal focus types. As the mapping between pitch accent categories and pitch types is not one-to-one, even in these speakers, the model also accounts for the proportions of falling and rising accents.

ii. The model can also account for the patterns of those speakers that use rising accents (almost) exclusively.

Crucially, the direction of the scaling from broad focus through narrow focus to contrastive focus is the same in both of the speaker groups.

In sum, although the two groups of speakers differ in their strategies for intonational marking of focus structure, their phonologies do not have to be completely different. Whereas in symbol-based phonological approaches we need to draw a line between speakers who choose different accent types and those simply varying phonetic parameters within a specific accent type, the approach put forward here treats both strategies as the output of the same dynamical system. The difference is captured simply by varying how each group of speakers makes use of the control parameter value space.

The model outlined in this paper characterises the form-function mapping of prosody and pragmatic meaning as flexible. While this mapping is not fixed, some important relations are always maintained: In the output of the system, higher proportions of rising accents will always co-occur with slightly larger rises (larger positive onglides). What is more, when two seemingly very different speaker strategies are compared, the model predicts modifications in the same direction when going from broad focus to narrow focus to contrastive focus as a consequence of the scaling of the control parameter (decreasing number of falls, increasingly large rises). The behaviour of a hypothetical group of speakers that uses falls in contrastive focus but rises in broad focus, for example, or small positive onglides in contrastive focus but large positive onglides in broad focus would not be captured by the present model.

The dynamical system used here is in fact a stochastic model. Thus, the data we observe are viewed as the outcome of a probabilistic process. The aim of this study was to present a possible model for the patterns we observe in the data. This system is certainly not the only possible model to capture a probabilistic mapping between forms and functions of intonation. Because the data exhibits two modes, it could as well be treated as the outcome of two independent probabilistic processes. However, we observe a trade-off between the two modes: When the rising mode grows, the falling mode shrinks. Our model presents one way of explaining this

dependence as the consequence of a dynamical landscape with two attractors. The attractors are not independently modulated, their respective stabilities are always linked through the scaling of the control parameter. While the model is able to describe this trade-off between the two modes in the data appropriately, this does not mean that it is the only possible way to explain this pattern of interdependence.

Current approaches to higher order dynamics of prosodic planning have already incorporated prosodic gestures (π gesture,μ gesture) into task dynamics [61,62]. These gestures modulate articulatory gestures relating to vowels and consonants at prosodic boundaries and on prosodically prominent constituents. They do not modulate parameters related directly to intonation like the model outlined in this work does. However, it should be emphasised that the present model is not able to capture all aspects of the production of intonation contours, especially the biophysical aspects (like task dynamics [63,64], or embodied task dynamics: [65]; see also [66,67] for specific models of vocal fold movements). The current model of intonation contours is only partial, being restricted to one parameter (onglide). In a full model, the state space would include all relevant parameters. In principle, a dynamical approach is not restricted to one control parameter–a full model with all relevant dimensions might also be characterised by multiple control parameters.

Our work has dealt with modelling of the production of intonationally marked focus structure, but to accurately model communication it is also necessary to take into account whether listeners perceive the focus structures as they are intended. One of the most interesting questions that arises from this research is whether both speaker groups would be perceived equally well in a perception experiment. Whereas one might assume that the "categorical" speakers in group 1 would yield more accurate perception scores, an earlier study by [68] on the perception of intonationally marked focus structures has indicated that this might not be the case. The authors subjected listeners to focus structures marked intonationally by different speakers. They found that all speakers were perceived similarly on average with no clear preference for "categorical" speakers. Moreover, they found interacting speaker-specific and listener-specific strategies. Thus, the same speaker could be perceived better than average by one listener but worse than average by another. Future work is needed to investigate how listeners "tune in" to a given speaker's productions. Although we showed an attractor landscape for production, it is also possible to model perception with attractor landscapes. The process of tuning in can be imagined as incrementally reorganising the attractor landscape based on the speaker's productions. Which processes facilitate the reorganisation of the attractor landscape is an open question for further research.

Overall, our model is able to account for the behavioural data fairly accurately. However, there are some shortcomings. For example, the differences between the medians of the group simulations are somewhat smaller than observed in the real data. It should be emphasised again that we are only using one phonetic dimension, namely the tonal onglide, to capture the categorical and continuous aspects of intonation which is clearly an oversimplification. Phonological categories are multi-dimensional [7,69–71], and intonational categories are no exception (for Italian and German: [72]; for German: [68,73]; for Italian: [30]; for English: [74], inter alia). The tonal onglide already indirectly captures some of the variation in two dimensions: the height of the f0 target and a binary measure of the alignment of the peak (falling onglides indicate that the peak is before the accented syllable and the target is fairly low, rising onglides indicate that the peak is within or shortly after the accented syllable and the target is high). As stated above, as the number of dimensions captured by the model increases, so will the complexity of the model. With regard to the number of attractors, it is also likely that more dimensions will contribute to a more complex attractor landscape–leading to a rich structure within each pitch accent type: While on the level of the onglide we might only need

two attractors, the level of the alignment might be characterised by three attractors constructing a landscape that could account for a potential differentiation between early, medial and late peak accents (H+!H*, H* and L+H*).

The dimensions in which pitch accents vary can be exploited differently by different speakers [72]. [35] looked at tonal onglide, target height and alignment in their data set and showed that while some speakers make little or no distinction in alignment between three focus conditions (broad, narrow, contrastive focus), they manipulate other parameters (target height, onglide) to a greater extent. In the current study, we were also able to identify different strategies in the use of the tonal onglide (i.e. group 1 uses falling *and* rising, while group 2 almost only uses rising accents). Apart from the other phonetic parameters that characterise pitch accents, aspects of the prenuclear contour may as well play an important role and need to be included in a complete modelling account. In addition to multiple dimensions that are associated with the structure of pitch accent categories, focus is also expressed by means of articulatory effort, varying on the hypo-hyper-articulation continuum [27,75–77]. These are also dimensions that could be integrated into a dynamical account of focus marking.

We have made first steps to model pitch accent types in a dynamical system. In doing so, we have extended the work on dynamical systems in phonetics and phonology to intonation research. Our work has applied this theoretical framework as an a posteriori assessment. Future research can derive hypotheses to test predictions of the model (or a similar model) and investigate important features of dynamical systems, like hysteresis and bifurcation, to consolidate the idea of intonation patterns as the result of a dynamical system.

5. Conclusion

This work reported on a controlled production study on German focus intonation and outlined a dynamical model to account for the phonological and phonetic variation found in the data. The model is able to unify both the categorical as well as the continuous aspects of pitch accents on one level, and offers a powerful tool to integrate the phonology and phonetics of prosodic patterns. Future research will explore how the perception of pitch accent categories can be modelled in a dynamical framework and how the inherent multi-dimensionality of phonological categories can be incorporated.

Supporting information

S1 Table. Target words used in the experiment (all nonce words).
(PDF)

S2 Table. Tools used in the experiment with English translation.
(PDF)

S1 Fig. Distance between real and simulated data for each tested k value for the data of all speakers and both groups. The dashed vertical line represents the k value with minimum distance.
(TIFF)

Acknowledgments

The authors thank Bodo Winter for his valuable advice on this research, Timo B. Roettger for his input on statistics and Lena Pagel for her help with the experiment.

Author Contributions

Conceptualization: Simon Roessig, Doris Mücke, Martine Grice.

Data curation: Simon Roessig.

Formal analysis: Simon Roessig.

Funding acquisition: Doris Mücke.

Investigation: Simon Roessig, Doris Mücke.

Methodology: Simon Roessig, Doris Mücke, Martine Grice.

Project administration: Doris Mücke.

Software: Simon Roessig.

Supervision: Doris Mücke, Martine Grice.

Visualization: Simon Roessig.

Writing – original draft: Simon Roessig.

Writing – review & editing: Doris Mücke, Martine Grice.

References

1. Pierrehumbert J. Phonological Representation: Beyond Abstract Versus Episodic. Annu Rev Linguist. 2016; 2:33–52.
2. Pierrehumbert J, Beckman ME, Ladd DR. Conceptual foundations of phonology as a laboratory science. In: Burton-Roberts N, Carr P, Docherty G, editors. Phonological knowledge: Conceptual and empirical issues. Oxford: Oxford University Press.; 2000. p. 273–304.
3. Ladd DR. Phonetics in Phonology. In: Goldsmith J, Riggle J, Yu ACL, editors. The Handbook of Phonological Theory. Malden: Blackwell; 2011. p. 348–73.
4. Ohala J. There is no interface between phonology and phonetics: A personal view. J Phon. 1990; 18:153–71.
5. Gafos AI. Dynamics in Grammar. In: Goldstein ML, Whalen DH, Best C, editors. Laboratory phonology 8: Varieties of Phonological Competence. Berlin, New York: Mouton de Gruyter; 2006. p. 51–79.
6. Gafos AI, Benus S. Dynamics of Phonological Cognition. Cogn Sci. 2006; 30(5):905–43. https://doi.org/10.1207/s15516709cog0000_80 PMID: 21702841
7. Mücke D. Dynamische Modellierung von Artikulation und prosodischer Struktur: Eine Einführung in die Artikulatorische Phonologie. Berlin: Language Science Press; 2018.
8. Fodor JA. The Language of Thought. Harvard University Press; 1975.
9. Fodor JA, Pylyshyn ZW. How Direct is Visual Perception? Some Reflections on Gibson's "Ecological Approach." Cognition. 1981; 9(2):139–96. PMID: 7196823
10. Newell A, Simon HA. Computer Science as Empirical Inquiry: Symbols and Search. Commun Assoc Comput Mach. 1981; 19:113–26.
11. Harnad S. The symbol grounding problem. Phys D Nonlinear Phenom. 1990; 42(1):335–46.
12. Spivey M. The Continuity of Mind. New York: Oxford University Press; 2007.
13. Kelso JA. Dynamic patterns: The self-organization of brain and behavior. Cambridge: MIT Press; 1995.
14. van Gelder T, Port R. Mind As Motion. MIT Press; 1995.
15. Port R, O'Dell M. Neutralization of syllable-final voicing in German. J Phon. 1985; 13:455–71.
16. Port R, Crawford P. Incomplete neutralization and pragmatics in German. J Phon. 1989; 17(4):257–82.
17. Ernestus M, Baayen H. The functionality of incomplete neutralization in Dutch: The case of past-tense formation. In: Goldstein ML, Whalen DH, Best C, editors. Laboratory phonology 8: Varieties of Phonological Competence. Berlin, New York: Mouton de Gruyter; 2006. p. 27–49.
18. Roettger TB, Winter B, Grawunder S, Kirby J, Grice M. Assessing incomplete neutralization of final devoicing in German. J Phon. 2014; 43:11–25.

19. Gussenhoven C. The phonology of tone and intonation. Cambridge: Cambridge University Press; 2004.
20. Beckman ME, Hirschberg J, Shattuck-Hufnagel S. The Original ToBI System and the Evolution of the ToBi Framework. In: Jun SA, editor. Prosodic Typology: The Phonology of Intonation and Phrasing. 2005.
21. Silverman K, Beckman M, Pitrelli J, Ostendorf M, Wightman CC, Price P, et al. TOBI: A Standard for Labeling English Prosody. In: Second International Conference on Spoken Language Processing. 1992.
22. Ladd DR, Morton R. The perception of intonational emphasis: Continuous or categorical? J Phon. 1997; 25:313–42.
23. Kügler F, Gollrad A. Production and perception of contrast: The case of the rise-fall contour in German. Front Psychol. 2015; 6:1254. https://doi.org/10.3389/fpsyg.2015.01254 PMID: 26388795
24. Grice M, Baumann S. Deutsche Intonation und GToBI. Linguist Berichte. 2002; 191:267–98.
25. Grabe E. Pitch accent realization in English and German. J Phon. 2004; 26(2):129–143.
26. Yoon T-J. Speaker consistency in the realization of prosodic prominence in the Boston University Radio Speech Corpus. In: Proceedings of Speech Prosody. Chicago; 2010.
27. Mücke D, Grice M. The effect of focus marking on supralaryngeal articulation—Is it mediated by accentuation? J Phon. 2014; 44:47–61.
28. Baumann S, Röhr C, Grice M. Prosodische (De-)Kodierung des Informationsstatus im Deutschen. Zeitschrift für Sprachwiss. 2015; 34:1–42.
29. Savino M, Grice M. The perception of negative bias in Bari Italian questions. In: Gorka E, Pietro P, editors. Prosodic Categories: Production, Perception and Comprehension. 2011. p. 187–206.
30. Cangemi F, Grice M. The Importance of a Distributional Approach to Categoriality in Autosegmental-Metrical Accounts of Intonation. Lab Phonol J Assoc Lab Phonol. 2016; 7(1):1–20.
31. Boersma P. Hayes B. Empirical Test of the Gradual Learning Algorithm. Linguist Inq. 2001; 32(1):45–86.
32. Coetzee AW, Anttila A. Integrating Variation in Phonological Analysis. In: Cohn A, Fougeron C, Huffmann MK, editors. The Oxford Handbook of Laboratory Phonology. 2012.
33. Ladd DR. Simultaneous structure in phonology. Oxford: Oxford University Press; 2014.
34. Bolinger D. Generality, gradience, and the all-or-none. Mouton Gravenhage; 1961.
35. Grice M, Ritter S, Niemann H, Roettger TB. Integrating the discreteness and continuity of intonational categories. J Phon. 2017; 64:90–107.
36. Tuller B, Case P, Ding M, Kelso JAS. The nonlinear dynamics of speech categorization. J Exp Psychol Hum Percept Perform. 1994; 20(1):3–16. PMID: 8133223
37. Mücke D, Hermes A, Cho T. Mechanisms of regulation in speech: Linguistic structure and physical control system. J Phon. 2017 Sep 1; 64:1–7.
38. Tuller B, Lancia L. Speech dynamics: Converging evidence from syllabification and categorization. J Phon. 2017; 64:21–33.
39. Nava E. Connecting Phrasal and Rhythmic Events: Evidence from Second Language Acquisition. Ph. D. dissertation, University of Southern California; 2010.
40. Iskarous K. The relation between the continuous and the discrete: A note on the first principles of speech dynamics. J Phon. 2017; 64:8–20.
41. Haken H, Levi P. Synergetic Agents: From Multi-Robot Systems to Molecular Robotics. Synergetic Agents: From Multi-Robot Systems to Molecular Robotics. Weinheim: Wiley; 2012.
42. Haken H, Kelso JAS, Bunz H. A theoretical model of phase transitions in human hand movements. Biol Cybern. 1985 Feb; 51(5):347–56. PMID: 3978150
43. Hock HS, Schöner G, Giese M. The dynamical foundations of motion pattern formation: Stability, selective adaptation, and perceptual continuity. Percept Psychophys. 2003 Apr; 65(3):429–57. PMID: 12785073
44. Lancia L, Winter B. The interaction between competition, learning, and habituation dynamics in speech perception. Lab Phonol. 2013; 4:221–58.
45. Tuller B, G. Jantzen M, K. Jirsa V. A dynamical approach to speech categorization: Two routes to learning. New Ideas Psychol. 2008; 26:208–26.
46. Thelen E, Smith LB. A dynamic systems approach to the development of cognition and action. Cambridge: The MIT Press; 1994.
47. Smith LB, Thelen E. Development as a dynamic system. Trends in Cognitive Sciences. 2003.

48. Mirman D, Magnuson JS. Dynamics of activation of semantically similar concepts during spoken word recognition. Mem Cognit. 2009 Oct; 37(7):1026–39. https://doi.org/10.3758/MC.37.7.1026 PMID: 19744941

49. Van Lieshout P. Dynamical Systems Theory and its application in speech. In: Maasen B, Kent R, Peters H, Van Lieshout P, Hulstijn W, editors. Oxford: Oxford University Press; 2004. p. 51–81.

50. Browman CP, Goldstein L. Articulatory phonology: an overview. Phonol Yearb. 1986; 3:219–52.

51. Haken H. Synergetics. In Berlin: Springer; 1977.

52. Ritter S, Grice M. The Role of Tonal Onglides in German Nuclear Pitch Accents. Lang Speech. 2015; 58(1):114–128.

53. Ladd DR. Intonational Phonology. Cambridge: Cambridge University Press; 2008.

54. Boersma P, Weenink D. Praat: doing phonetics by computer [Internet]. 2018. Available from: http://www.praat.org/

55. Löfqvist A, Baer T, McGarr NS, Story RS. The cricothyroid muscle in voicing control. J Acoust Soc Am. 1989 Mar; 85(3):1314–21. PMID: 2708673

56. R Core Team. R: A Language and Environment for Statistical Computing [Internet]. Vienna, Austria; 2018. Available from: http://www.r-project.org/

57. Bürkner P-C. Advanced Bayesian Multilevel Modeling with the R Package brms. R J. 2018;

58. Carpenter B, Gelman A, Hoffman M, Lee D, Goodrich B, Betancourt M, et al. Stan: A Probabilistic Programming Language. J Stat Software, Artic. 2017; 76(1):1–32.

59. Brown SD, Ratcliff R, Smith PL. Evaluating methods for approximating stochastic differential equations. J Math Psychol. 2006 Aug; 50(4):402–410. https://doi.org/10.1016/j.jmp.2006.03.004 PMID: 18574521

60. Gelder T Van Port R. It's About Time: An Overview of the Dynamical Approach to Cognition. In: Mind as motion: Explorations in the dynamics of cognition. 1995.

61. Byrd D, Saltzman E. The elastic phrase: modeling the dynamics of boundary-adjacent lengthening. J Phonetics. 2003; 31:149–80.

62. Saltzman E, Nam H, Krivokapic J, Goldstein L. A task-dynamic toolkit for modeling the effects of prosodic structure on articulation. In: Proceedings of the 4th International Conference on Speech Prosody, Campinas. 2008. p. 175–174.

63. Saltzmann E. Task dynamic coordination of the speech articulators: a preliminary model. In: Heuer H, Fromm C, editors. Generation and Modulation of Action Patterns. Berlin: Springer; 1986. p. 129–144.

64. Hawkins S. An introduction to task dynamics. In: Ladd DR, Docherty GJ, editors. Gesture, Segment, Prosody. Cambridge: Cambridge University Press; 1992. p. 9–25. (Papers in Laboratory Phonology).

65. Simko J, Cummins F. Embodied task dynamics. Psychol Rev. 2010; 117(4):1229–46. https://doi.org/10.1037/a0020490 PMID: 21038977

66. Story BH, Titze IR. Voice simulation with a body-cover model of the vocal folds. J Acoust Soc Am. 1995; 97(2):1249–60. PMID: 7876446

67. Zhang Z. Effect of vocal fold stiffness on voice production in a three-dimensional body-cover phonation model. J Acoust Soc Am. 2017; 142(4):2311. https://doi.org/10.1121/1.5008497 PMID: 29092586

68. Cangemi F, Krüger M, Grice M. Listener-Specific Perception of Speaker-Specific Productions in Intonation. In: Fuchs S, Pape D, Petrone C, Perrier P, editors. Individual Differences in Speech Production and Perception. Frankfurt a. M.: Peter Lang; 2015.

69. Lisker L. "Voicing" in English: A Catalogue of Acoustic Features Signaling /b/ Versus /p/ in Trochees. Lang Speech. 1986; 29:3–11. https://doi.org/10.1177/002383098602900102 PMID: 3657346

70. Coleman J. Discovering the acoustic correlates of phonological contrasts. J Phon. 2003; 31:351–72.

71. Winter B. Prospects & Overviews Spoken language achieves robustness and evolvability by exploiting degeneracy and neutrality. Bioessays. 2014; 36:960–7. https://doi.org/10.1002/bies.201400028 PMID: 25088374

72. Niebuhr O, D'Imperio M, Gili Fivela B, Cangemi F. Are There "Shapers" and "Aligners"? Individual Differences in Signalling Pitch Accent Category. In: Proceedings of the 17th ICPhS. Hong Kong; 2011. p. 120–123.

73. Ambrazaitis G, Frid J. F0 Peak Timing, Height, and Shape as Independent Features. In: Gussenhoven C, Chen Y, Dediu D, editors. Proc of The 4th International Symposium on Tonal Aspects of Languages, Nijmegen, The Netherlands. ISCA; 2014. p. 138–42.

74. Barnes J, Veilleux N, Brugos A, Shattuck-Hufnagel S. Tonal Center of Gravity: A global approach to tonal implementation in a level-based intonational phonology. Lab Phonol. 2012; 3:337–83.

75. Harrington J, Fletcher J, Beckman ME. Manner and place conflicts in the articulation of accent in Australian English. In: Broe M, editor. Papers in laboratory phonology V: Acquisition and the Lexicon. Cambridge: Cambridge University Press; 2000. p. 40–51.

76. de Jong KJ. The supraglottal articulation of prominence in English: linguistic stress as localized hyperarticulation. J Acoust Soc Am. 1995 Jan; 97(1):491–504. PMID: 7860828

77. Cho T, McQueen J. Prosodic influences on consonant production in Dutch: Effects of prosodic boundaries, phrasal accent and lexical stress. J Phon. 2005; 33(2):121–57.
